# Direct AMPK Activation Confers Mutation‐Independent Therapeutic Benefit in Duchenne Muscular Dystrophy

**DOI:** 10.1002/jcsm.70200

**Published:** 2026-02-04

**Authors:** Sean Y. Ng, Andrew I. Mikhail, Stephanie R. Mattina, Magda A. Lesinski, Irena A. Rebalka, Sophie I. Hamstra, Donald Xhuti, Val A. Fajardo, Mark A. Tarnopolsky, Joshua P. Nederveen, Gregory R. Steinberg, Thomas J. Hawke, Vladimir Ljubicic

**Affiliations:** ^1^ Department of Kinesiology McMaster University Hamilton Ontario Canada; ^2^ Department of Pathology and Molecular Medicine McMaster University Hamilton Ontario Canada; ^3^ Centre for Metabolism, Obesity and Diabetes Research McMaster University Hamilton Ontario Canada; ^4^ Department of Kinesiology Brock University St. Catharines Ontario Canada; ^5^ Department of Pediatrics McMaster University Medical Centre Hamilton Ontario Canada; ^6^ Department of Medicine, Division of Endocrinology and Metabolism McMaster University Hamilton Ontario Canada; ^7^ Department of Biochemistry and Biomedical Sciences McMaster University Hamilton Ontario Canada

**Keywords:** AMP‐activated protein kinase, Duchenne muscular dystrophy, heart, mitochondria, neuromuscular junction, skeletal muscle, utrophin

## Abstract

**Background:**

Duchenne muscular dystrophy (DMD) is a severe, life‐limiting neuromuscular disorder (NMD) characterized by progressive muscle wasting and mitochondrial dysfunction. Although gene therapies offer promise, even those already approved by regulatory agencies, their use remains constrained by mutation specificity, delivery challenges and durability. Pharmacologically targeting AMPK has shown potential to ameliorate dystrophic pathology, but prior strategies have been hindered by inadequate efficacy and off‐target effects.

**Methods:**

Comparative transcriptomic analyses were conducted to assess concordance between gene expression profiles induced by direct AMPK activation and those observed in DMD patient muscle. To evaluate the therapeutic potential of sustained AMPK activation, DBA/2J‐mdx (D2.mdx; *n* = 8–10) mice were treated daily with MK‐8722 (MK; 5 mg·kg^−1^) or vehicle for 7 weeks, with healthy DBA/2J mice serving as controls. Additionally, DMD patient‐derived myotubes (*n* = 4) were treated with MK (1 μM, 24 h) to assess AMPK‐mediated cellular adaptations in human cells.

**Results:**

Transcriptomic profiling revealed that MK modulated 206 DMD‐associated transcripts, reversing expression of 73 upregulated and 133 downregulated genes. In vivo, MK‐treated D2.mdx mice showed enhanced AMPK signalling (ACC phosphorylation, +120%–150%; *p* < 0.05), increased *Ppargc1a* expression (+60%, *p* < 0.05) and reduced inflammation‐ and fibrosis‐associated transcripts (−25%–50%; *p* < 0.05). Seven weeks of daily MK treatment enhanced (+95%; *p* < 0.05) whole‐body lipid oxidation during active periods without affecting energy expenditure or activity. Notably, we observed that repeated MK dosing did not adversely impact cardiac morphology or function (*p* > 0.05). MK‐treated D2.mdx mice demonstrated improved grip strength fatigability (−35%, *p* < 0.05), inverted hang performance (+100%, *p* < 0.05) and treadmill exercise capacity (+20%, *p* < 0.05). Additional ex vivo muscle assessments revealed (+30%; *p* < 0.05) greater peak isometric force and improved twitch contractile kinetics. These functional adaptations were coincident with reduced myofibre damage and fibrosis, with increased sarcolemmal expression of utrophin, γ‐sarcoglycan and β‐dystroglycan (+10%–70%; *p* < 0.05), despite no changes in fibre size or mass. Mitochondrial assessments showed increased State III respiration (+80%; *p* < 0.05), reduced reactive oxygen species production (−70%; *p* < 0.05) and elevated OXPHOS protein content (+25%–45%; *p* < 0.05). In patient‐derived myotubes, MK similarly activated AMPK signalling, increased mitochondrial electron transport chain proteins (+25%–35%; *p* < 0.05) and enhanced maximal oxygen consumption (+50%–80%; *p* < 0.05) in DMDΔ44 and DMDΔ45 lines.

**Conclusions:**

These findings demonstrate that sustained, systemic activation of AMPK safely improves muscle function, metabolic health and dystrophic pathology in a mutation‐independent manner. This supports the therapeutic potential of direct AMPK agonists as a disease‐modifying strategy for DMD and other neuromuscular disorders.

## 1 Introduction

Duchenne muscular dystrophy (DMD) is a severe X‐linked recessive disorder caused by mutations in the DMD gene, which encodes the sarcolemmal protein dystrophin [1, 2]. The absence or near‐complete deficiency of dystrophin renders muscle fibres vulnerable to repeated cycles of damage and incomplete repair, ultimately leading to progressive muscle degeneration and premature mortality, often due to respiratory or cardiac failure [1]. Despite advances in understanding the genetic basis of DMD and the emergence of several recently approved dystrophin‐targeted therapies [3, 4], effective long‐term treatments that broadly address the multifactorial pathology of DMD remain limited. This is particularly problematic given the heterogeneity in disease progression and the wide spectrum of DMD mutations, which constrain the applicability of mutation‐specific strategies [1, 5]. Moreover, many gene‐based approaches face challenges related to delivery, immunogenicity, durability and cost. These issues highlight the need for accessible, mutation‐independent interventions.

AMP‐activated protein kinase (AMPK) is a highly conserved energy sensor that regulates key processes essential for skeletal muscle health, including autophagy, mitochondrial biogenesis and neuromuscular remodelling [6, 7, 8, 9, 10]. Work from Bernard Jasmin's laboratory was the first to demonstrate that pharmacological stimulation of AMPK confers therapeutic benefit in mdx mice [11]. These findings have since been supported by a growing body of preclinical evidence indicating that enhancing AMPK signalling mitigates several pathological features of dystrophic muscle, such as inflammation, fibrosis and impaired mitochondrial metabolism [12, 13, 14, 15, 16, 17, 18, 19]. AMPK activation has also been shown to increase utrophin expression and promote its distribution along the sarcolemma, a well‐established mutation‐independent mechanism for reinforcing membrane integrity in dystrophic muscle. However, translation of these findings into the human DMD condition has been hampered by several limitations associated with the modalities available to stimulate AMPK, such as deleterious off‐target effects and low bioavailability. These issues arise, at least in part, from the indirect mechanisms of AMPK agonism used by compounds like metformin (MET) and resveratrol and the pleiotropic effects observed with nucleotide analogs such as AICAR [20].

Several next‐generation AMPK activators have been recently identified with improved specificity, safety and efficacy. Among these, MK‐8722 (MK) is a well‐characterized, orally bioavailable direct AMPK agonist that binds the allosteric drug and metabolite site at the interface of the AMPK α and β subunits and leads to potent activation of AMPK heterotrimers containing either the β1 or β2 isoforms. The β2 isoform is predominantly expressed in skeletal muscle, rendering MK particularly effective in targeting muscle‐specific AMPK signalling. As such, it has shown promising efficacy in neuromuscular disease models, including DMD and myotonic dystrophy Type I mice [10, 21, 22]. Thus, MK serves as a useful pharmacological tool to probe the therapeutic potential of direct AMPK agonism in neuromuscular conditions.

In the present study, we further explored the role of AMPK in DMD by testing the efficacy and safety of direct, pharmacological AMPK activation. We hypothesized that (1) pharmacological induction of AMPK would engage conserved cellular stress and metabolic pathways disrupted in DMD; (2) chronic stimulation of AMPK via daily MK administration to D2.mdx mice would ameliorate key features of DMD pathology in skeletal muscle without inducing adverse outcomes; and (3) these benefits would be recapitulated in multiple human DMD muscle cell lines. Our findings show that long‐term exposure to MK administration safely attenuates dystrophic pathology and enhances mitochondrial biology in severe DMD mice. Parallel benefits were observed in DMD patient‐derived myotubes harbouring diverse mutations, thereby supporting the mutation‐independent therapeutic potential of direct AMPK activation.

## 2 Methods

### 2.1 Animal Experiments

Five‐week‐old male *D2.mdx* and *DBA/2J WT* mice (Jackson Laboratory) were housed under standard conditions. Mice were treated with an AMPK agonist (MK, 5 mg·kg^−1^) or vehicle (Veh) via oral gavage. Acute MK effects were assessed at 1, 3 and 12 h post‐dose. Chronic treatment was delivered daily for 7 weeks. Body composition (fat and lean mass) was assessed via quantitative NMR, and whole‐body metabolism was evaluated using metabolic cages. Ambulatory activity was analysed using open‐field monitoring, whereas in vivo muscle function was assessed through grip strength, hanging endurance and treadmill running capacity. Cardiac structure and function were evaluated via transthoracic echocardiography using M‐mode and Doppler imaging. At the end of the experimental period, animals were euthanized, and skeletal muscles were harvested for ex vivo muscle contractility, high‐resolution respirometry, muscle histology, immunofluorescence, immunoblotting and ultrastructure evaluation of mitochondria. Full and detailed protocols are provided in the Supplementary Methods.

### 2.2 Patient‐Derived Myoblast Culture and MK Treatment

Human myoblasts were derived from skeletal muscle biopsies of DMD patients (Fondazione IRCCS Carlo Besta; see Table S1). Cells were cultured under standard growth and differentiation conditions. DMD myoblasts were treated with vehicle or MK (1 or 5 μM) for 24 h. Cells were then processed for immunoblotting or Seahorse metabolic analysis. Detailed media formulations and dosing protocols are provided in the Supplementary Methods.

### 2.3 Bioinformatic Analysis of Transcriptomic Datasets

Differentially expressed genes (DEG) in skeletal muscle from DMD patients compared to healthy paediatric controls were identified previously (GSE38417), with statistical significance reported in the original studies [23]. DEGs in response to AMPK agonists or exercise in C57BL/6 mice (GSE92719 [24]) were subsequently analysed using Geo2R, with statistical significance defined as *p* < 0.05 via limma moderated *t*‐tests. Select mitochondrial or extracellular matrix (ECM) heatmaps were generated by manually calculating Z‐scores from the GSE3307 dataset and visualized using GraphPad Prism.

### 2.4 Statistical Analyses

In the acute MK experiment, one‐way ANOVA and Tukey post hoc tests were utilized to identify significant differences. For the chronic experiments, a one‐way ANOVA and Tukey post hoc tests were performed to examine the differences among groups. Hanging and running survival data from these animals were evaluated using the log‐rank test to identify any significant differences. To determine significant changes on gene expression in the acute experiment, an unpaired *t*‐test was employed. GraphPad Prism software (V9.1.1; San Diego, CA, USA) was used for statistical analysis. All individual data points are displayed. Data are expressed as mean ± SEM. Statistical significance was accepted at *p* < 0.05.

## 3 Results

### 3.1 A Single Dose of AMPK Activation Affects the Dystrophic Gene Signature

**FIGURE 1:**
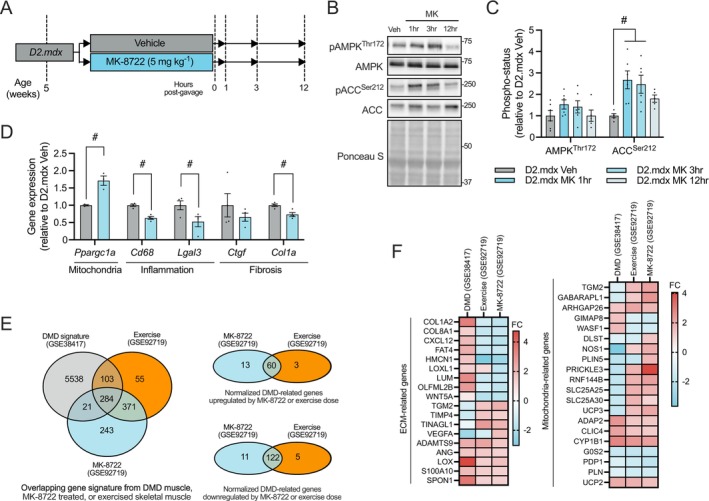
A single dose of AMP‐activated protein kinase (AMPK) activation affects the dystrophic gene signature. (A) Experimental design to investigate the effects of a single dose of MK‐8722 (MK) in D2.mdx animals. Five‐week‐old male DBA/2J‐mdx (D2.mdx) mice received an oral dose of vehicle (Veh) or MK solution (5 mg·kg^−1^). MK‐treated mice were euthanized 1‐, 3‐ or 12‐h post‐gavage. Veh‐treated mice were euthanized at the same time points and pooled as a control group. (B) Representative western blots for threonine 172 (Thr^172^)‐phosphorylated AMPK (pAMPK^Thr172^), AMPK, Ser212 (Ser^212^)‐phosphorylated acetyl‐CoA carboxylase (pACC^Ser212^) and ACC in the tibialis anterior (TA) muscles of Veh‐ and MK‐treated D2.mdx mice. Ponceau S staining shows equal loading. Protein ladder values (kDa) are indicated on the right. (C) Graphical summaries of AMPK and ACC phosphorylation status (i.e., phospho/total protein ratio) in TA muscles of Veh‐ or MK‐treated mice. (D) Graphical summaries of mitochondria (Mito)‐, inflammation‐ and fibrosis‐related genes in the TA muscles of D2.mdx Veh and D2.mdx MK 3‐h mice. Data are presented as individual points, and group means (bars) with standard error means (SEM) (*n* = 3–6). (E) Venn diagram showing the overlap between DMD patient skeletal muscle gene signature (GSE38417) and muscle genes regulated by a single dose of exercise or MK treatment (GSE92719) in mice. To the right, Venn diagrams of differentially expressed genes in DMD patient muscle that are normalized by exercise or MK‐mediated downregulation or upregulation. (F) Heat maps of transcripts from gene ontologies related to extracellular matrix (ECM) and mitochondria. Statistical significance is denoted as follows: #, *p* < 0.05 versus Veh‐treated D2.mdx mice.

To investigate the impact of targeted AMPK activation in a clinically relevant and severely dystrophic skeletal muscle, we administered MK (5 mg·kg^−1^) via oral gavage to D2.mdx animals. Tibialis anterior (TA) muscles were collected 1, 3 and 12 h after a single dose of either Veh or MK to assess skeletal muscle AMPK activation and downstream signalling germane to the DMD phenotype (Figure 1A). Immunoblotting analyses revealed that AMPK threonine 172 phosphorylation status (i.e., phosphorylated form/total protein ratio) was similar (*p* > 0.05) across all timepoints following MK (Figure 1B,C). In contrast, acetyl‐CoA carboxylase (ACC) serine 212 phosphorylation status was 120%–150% higher (*p* < 0.05) in the 1‐ and 3‐h MK groups when compared to their Veh‐treated counterparts (Figure 1B,C). We then examined AMPK‐related mRNA levels in the TA muscles of dosed D2.mdx animals at 3‐h post‐treatment to capture early transcriptional responses. A 60% increase (*p* < 0.05) in *Ppargc1a* transcript levels was detected in the MK‐treated D2.mdx mice relative to the Veh group (Figure 1D). Additionally, MK dosing significantly reduced expression of inflammation‐ and fibrosis‐related mRNAs *Cd68*, *Lgal3* and *Col1a* by 25%–50% (*p* < 0.05) compared to Veh controls (Figure 1D).

To contextualize these findings, we integrated transcriptomic data from DMD patient biopsies (GSE38417) and mouse AMPK activation datasets (GSE92719). We identified 284 overlapping genes, with 73 upregulated and 133 downregulated following MK (Figure 1E). Notably, 182 of these genes also responded to exercise and were enriched for ECM and mitochondrial gene ontologies (Figure 1F), highlighting shared transcriptional responses to MK and exercise. Together, these data validate pharmacological AMPK activation via MK in D2.mdx skeletal muscle and demonstrate the intersection between AMPK‐induced skeletal muscle plasticity and the pathomechanisms of DMD.

### 3.2 Daily Pharmacological AMPK Stimulation Enhances Whole‐Body Lipid Metabolism Without Altering Basal Activity Levels in D2.mdx Animals

**FIGURE 2:**
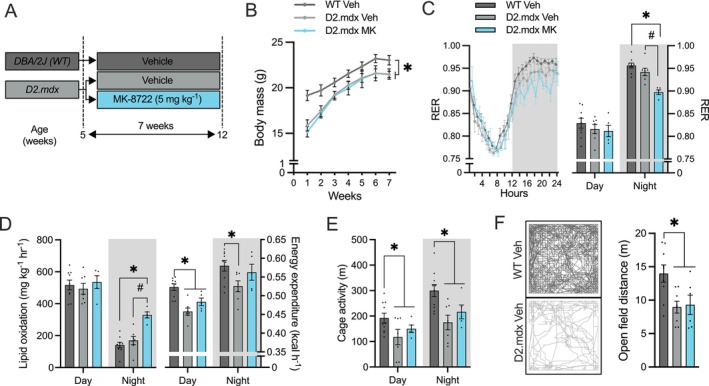
Daily pharmacological AMPK stimulation enhances whole‐body lipid metabolism without altering basal activity levels in D2.mdx animals. (A) Schematic of MK dosing regimen. Five‐week‐old D2.mdx mice received daily oral gavage with either Veh or MK (5 mg·kg^−1^) for 7 weeks. Veh‐treated DBA/2 J (wild‐type [WT]) mice served as healthy controls. (B) Body mass (g) trajectories of Veh‐ and MK‐treated animals throughout the 7‐week experimental period. (C) Average respiratory exchange ratio (RER) curves and graphical summary over 24 h in animals treated with Veh or MK for 7 weeks. (D) Lipid oxidation rates (mg·kg^−1^·h^−1^) and whole‐body energy expenditure (kilocalorie per hour; kcal·h^−1^) during the day and night periods from the three experimental groups. (E) Cumulative cage activity, expressed as distance travelled (m), during the day and night periods in mice treated with Veh or MK for 7 weeks. (F) Representative tracings of locomotor activity during a 10‐min open field test in Veh‐treated WT and D2.mdx animals, with a graphical summary on the right showing total distance travelled. Graphs display individual data points, group means (bars) and standard errors of the mean (SEM). *n* = 8–10. Statistical significance is denoted as follows: *, *p* < 0.05 versus WT Veh; #, *p* < 0.05 versus D2.mdx Veh.

We next evaluated whether long‐term AMPK activation would induce adaptive changes in D2.mdx mice. Accordingly, we treated 5‐week‐old D2.mdx mice with MK (5 mg·kg^−1^) orally each day for 7 weeks (Figure 2A). During this period, the D2.mdx Veh group displayed significantly lower body mass (−7.5%; Figure 2B) but greater body fat (+50%; *p* < 0.05, data not shown), compared to the healthy control condition. Following 7 weeks of MK dosing, D2.mdx mice displayed similar (*p* > 0.05) total body mass and body fat percentage when compared to their Veh‐treated counterparts.

Metabolic phenotyping revealed that the respiratory exchange ratio (RER) and lipid oxidation rates were similar (*p* > 0.05) among wild‐type (WT) and D2.mdx groups during the daylight periods. Evening measurements showed a significantly lower RER (Figure 2C) and higher (+95%; *p* < 0.05) lipid oxidation rates in MK‐treated D2.mdx animals compared to the D2.mdx Veh group (Figure 2D). Additional metabolic metrics indicated that energy expenditure was 10%–15% lower (*p* < 0.05; Figure 2D) and ambulatory activity reduced by 35%–40% (*p* < 0.05; Figure 2E,F) in D2.mdx animals irrespective of time of day. Repeated MK dosing had little to no effect on these measures. These findings suggest that daily AMPK activation enhances whole‐body metabolism in D2.mdx mice without altering activity levels.

### 3.3 Repeated AMPK Activation Does Not Exacerbate the DMD Cardiac Phenotype in D2.mdx Animals

Chronic transgenic or pharmacological activation of AMPK in mammals has been linked to deleterious off‐target effects, including cardiac dysfunction and hypertrophy [6, 25, 26]. Because AMPK‐mediated adaptations might overlap with complications observed in DMD hearts [27, 28], we sought to investigate whether repeated dosing with MK exacerbated dystrophic cardiac symptoms. To this end, following 7 weeks of Veh or MK dosing in D2.mdx mice, heart structure and function were examined in vivo using echocardiography. All cardiac morphology metrics were unaffected by chronic AMPK activation (Figure S1A–D). Cardiac function metrics were also similar (*p* > 0.05) between Veh and MK groups with the exception of peak mitral E‐wave velocity and A‐wave acceleration, which were increased by MK and similar (*p* > 0.05) to the WT Veh condition (Figure S1E–N). To assess whether MK influenced structural remodelling, we additionally quantified cardiac fibrosis, which was unaltered by MK treatment (Figure S1O–Q). On balance, these data indicate that repeated AMPK activation by MK did not change cardiac morphology or functional metrics in D2.mdx mice.

### 3.4 Repeated AMPK Activation Improves Skeletal Muscle Function in Severe DMD Mice

**FIGURE 3:**
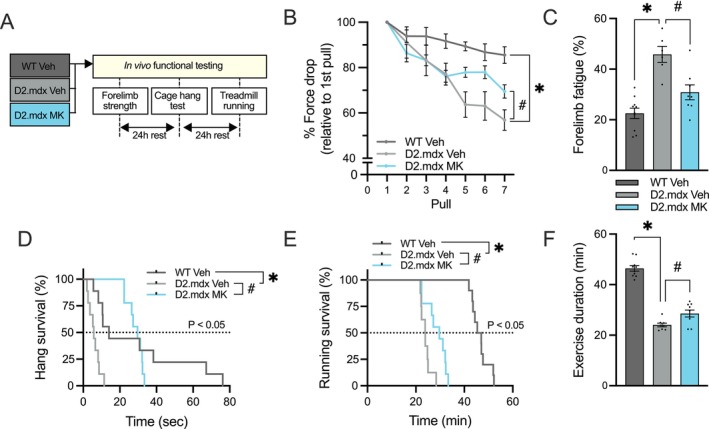
Repeated AMPK activation improves in vivo skeletal muscle function in severe DMD mice. (A) Schematic of in vivo muscle function assessments following long‐term treatment with Veh or MK. (B) Successive forelimb grip strength measurements expressed as a percentage of the first pull in Veh‐treated WT and Veh‐ or MK‐treated D2.mdx animals. (C) Forelimb fatigue index (% difference between first and last forelimb grip strength measures) in Veh‐treated WT and Veh‐ or MK‐treated D2.mdx animals. (D) Kaplan–Meier analysis of inverted cage hang time (s) in all experimental animals. (E) Kaplan–Meier analysis of exercise duration (min) during the treadmill running test in Veh‐treated WT and Veh‐ or MK‐treated D2.mdx animals. Both Kaplan–Meier plots display the number of animals remaining in the test as a percentage, with counts decreasing as events occurred during the assay. Median time to event is indicated by the dotted line. Log‐rank *p*‐values are reported for between‐group comparisons. (F) Time to exhaustion (min) during the exercise tolerance test across all groups. Graphs display individual data points, group means (bars) and SEM. *n* = 8–10. Statistical significance is denoted as follows: *, *p* < 0.05 versus WT Veh; #, *p* < 0.05 versus D2.mdx Veh.

To evaluate the skeletal muscle adaptations endowed by repeated MK activation in dystrophic mice, a series of in vivo muscle functional assessments were performed (Figure 3A). As expected, Veh‐treated D2.mdx animals exhibited a 25% reduction (*p* < 0.05) in maximum forelimb grip strength (data not shown) and a significant increase in forelimb fatigability (*p* < 0.05; Figure 3B,C) compared to their healthy WT counterparts. Although MK treatment did not alter absolute grip strength force in D2.mdx mice, forelimb fatigability was reduced by 35% (*p* < 0.05), reaching levels comparable to those seen in healthy controls (Figure 3B,C). Furthermore, the typical reduction (−75%; *p* < 0.05) in cage hang time (Figure 3D) and lower (−50%, *p* < 0.05) exercise tolerance (Figure 3E,F) observed in the D2.mdx animals were attenuated following repeated AMPK activation. MK‐treated D2.mdx mice displayed a twofold increase (*p* < 0.05) in cage hang performance and 20% greater (*p* < 0.05) exercise duration when compared to the D2.mdx Veh group.

**FIGURE 4:**
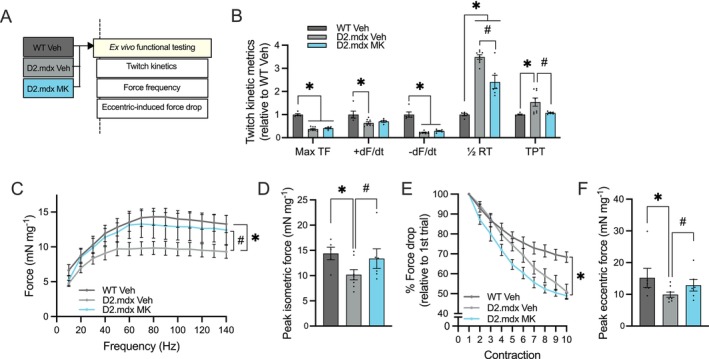
Repeated AMPK activation improves ex vivo skeletal muscle function in D2.mdx animals. (A) Schematic of ex vivo muscle function assessments following long‐term treatment with Veh or MK. (B) Summary of extensor digitorum longus (EDL) twitch kinetics expressed relative to the WT Veh group: maximal twitch force (Max TF), maximal rate of force development (+dF/dt), maximal rate of relaxation (−dF/dt), half relaxation time (½ RT) and time to peak tension (TPT). (C) Ex vivo force–frequency curves of the EDL muscles across all experimental groups. Force values are expressed relative to muscle mass (mN·mg^−1^). (D) Peak tetanic force (mN) of EDL muscles from Veh‐treated WT animals and Veh‐ and MK‐treated D2.mdx animals. (E) Relative force drop trace compared to the first eccentric contraction following 10 eccentric contractions across all groups. (F) Maximal force output during ex vivo EDL eccentric contractions, expressed relative to muscle mass (mN·mg^−1^). Graphs display individual data points, group means (bars) and SEM. *n* = 8–10. Statistical significance is denoted as follows: *, *p* < 0.05 versus WT Veh; #, *p* < 0.05 versus D2.mdx Veh.

Next, we sought to appraise MK‐mediated skeletal muscle adaptations through ex vivo assessment of the extensor digitorum longus (EDL) (Figure 4A). Muscle twitch kinetics were significantly altered in the EDL muscles of D2.mdx mice compared to WT controls (Figure 4B). MK‐treated D2.mdx animals showed a 30% reduced (*p* < 0.05) half relaxation time and a shorter time to peak tension (−30%; *p* < 0.05), with these values approaching those observed in the healthy WT group. Consistent with prior studies [29] and corroborating our in vivo data, electrically stimulated EDL muscle force in Veh‐treated D2.mdx animals was 30% lower (*p* < 0.05) compared to the healthy WT group (Figure 4C,D). D2.mdx MK mice demonstrated a 30% increase (*p* < 0.05) in relative peak isometric force production compared to D2.mdx Veh counterparts. Next, we stimulated the EDL muscle with a series of 10 eccentric contractions (ECCs). Dystrophic EDL muscles exhibited a 50% reduction (*p* < 0.05) in force between the first and last ECC, whereas WT Veh EDL muscles showed a typical 35% decline (Figure 4E). Repeated MK dosing led to significantly higher (+30%) peak eccentric force, reaching levels comparable to those of healthy controls (Figure 4F), and maintained a ~40% higher force relative to Veh‐treated mice even after the ECC protocol. Collectively, these in vivo and ex vivo muscle evaluations indicate that chronic AMPK activation improved dystrophic muscle function by attenuating fatigability, increasing exercise tolerance and partially restoring muscle force production to near‐healthy levels.

Given the observed improvements in muscle function in MK‐treated D2.mdx animals, we next investigated whether these adaptations might be driven by changes at the neuromuscular junction (NMJ). Whole‐mount staining of the epitrochleoanconeus (ETA) muscle and confocal microscopy revealed a fragmented NMJ morphology in D2.mdx animals, consistent with previous reports [30]. Repeated AMPK activation enhanced some NMJ postsynaptic features, including enlarged (+25%–30%; *p* < 0.05) acetylcholine receptor (AChR) and endplate areas (Figure S2A,B). These data suggest that MK treatment enhances NMJ stability in dystrophic skeletal muscle, potentially contributing to improved muscle performance.

### 3.5 AMPK Attenuates Skeletal Muscle Histopathology Independent of Muscle Morphology in D2.mdx Mice

**FIGURE 5:**
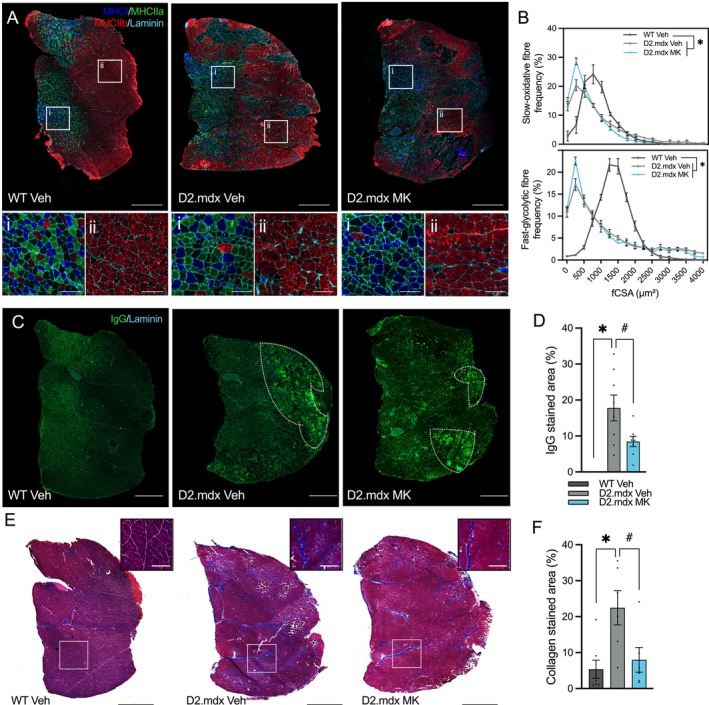
AMPK attenuates skeletal muscle histopathology independent of muscle morphology in D2.mdx mice. (A) Representative immunofluorescence (IF) microscopy images showing myosin heavy chain I (MHCI; blue), MHCIIa (green), MHCIIb (red) and laminin (cyan) in GAST muscles from WT Veh, D2.mdx Veh and D2.mdx MK mice. Enlarged insets highlight slow‐oxidative (SO; i) and fast‐glycolytic (FG; ii) regions of GAST muscle sections. Scale bars denote 1000 μm (whole muscle) and 100 μm (insets). (B) Fibre cross‐sectional area (fCSA, μm^2^) distribution in SO and FG regions of GAST muscles in Veh‐ and MK‐treated mice. (C) Representative IF images of immunoglobulin G (IgG; green) and laminin (cyan) staining in GAST muscles of Veh‐ and MK‐treated mice. Areas enclosed by the dotted lines indicate accumulations of IgG‐positive fibres. Scale bar: 1000 μm. (D) Graphical summary of IgG‐positive areas in GAST muscle cross‐sections of all groups. (E) Masson's trichrome staining of GAST muscle sections from WT Veh, D2.mdx Veh and D2.mdx MK mice. Insets show collagen deposits (blue) in the medial head of the GAST muscle. Scale bars: 1000 μm (whole muscle, black) and 200 μm (inset, white). (F) Graphical summary of collagen‐stained area in WT Veh, D2.mdx Veh and D2.mdx MK mice. Graphs show individual data points, group means (bars), and SEM. *n* = 8–10. Statistical significance is denoted as follows: *, *p* < 0.05 versus WT Veh; #, *p* < 0.05 versus D2.mdx Veh.

To accompany our assessment of the functional adaptations bestowed by MK, we next examined skeletal muscle mass and muscle fibre morphology. A typical reduction (−15%–20%) of skeletal muscle mass [triceps (TRI), quadriceps (QUAD), TA and gastrocnemius (GAST)] was observed in D2.mdx animals when compared to age‐matched healthy controls (Figure S3A). Similarly, the GAST muscle from D2.mdx mice displayed common dystrophic features, such as altered fibre type distribution (Figure 5A and Figure S3B), reduced muscle fibre size in both slow oxidative (SO) and fast glycolytic (FG) fibres (Figure 5B and Figure S3D) and increased fibre size variability (Figure S3E). Chronic MK treatment did not affect these morphological metrics.

D2.mdx Veh animals exhibited a significant increase (+15%) in intramyocellular IgG‐positive staining (Figure 5C,D), indicative of heightened muscle damage. Additionally, collagen accumulation was observed (+15%–25%; *p* < 0.05) throughout dystrophic GAST muscles, displaying substantial fibrosis relative to healthy controls (Figure 5E,F). D2.mdx animals treated with MK showed a 50% reduction (*p* < 0.05) in IgG‐positive staining and a 65% decrease (*p* < 0.05) in ECM deposition. Altogether, these histological findings demonstrate that chronic pharmacological AMPK activation does not prevent muscle fibre atrophy in dystrophic skeletal muscle but augments resilience to muscle damage and fibrosis.

### 3.6 Elevated Utrophin‐Associated Protein Complex Expression Following Pharmacological AMPK Stimulation in Severely Dystrophic Skeletal Muscle

**FIGURE 6:**
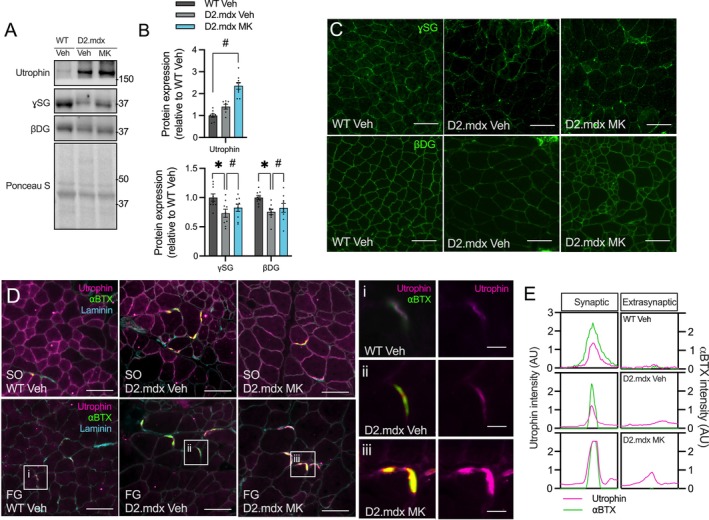
Elevated utrophin‐associated protein complex expression following pharmacological AMPK stimulation in severely dystrophic skeletal muscle. (A) Typical western blots showing utrophin, γ‐sarcoglycan (γSG) and β‐dystroglycan (βDG) in TA muscles from Veh‐treated WT, D2.mdx Veh and D2.mdx MK mice. Ponceau S staining serves as a loading control. Protein ladder markers are indicated in kDa. (B) Graphical summaries of utrophin, βDG and γSG content across all groups. Values are expressed relative to WT Veh. (C) Representative IF microscopy images of γSG and βDG in the FG regions of GAST muscles from all experimental groups. Scale bars: 100 μm. (D) Representative IF images showing utrophin (magenta) in the SO and FG regions of GAST muscles from Veh‐treated WT, D2.mdx Veh and D2.mdx MK mice. The postsynaptic compartment of the neuromuscular junction is marked by α‐bungarotoxin (αBTX; green), and the sarcolemma is outlined by laminin (cyan). Insets i, ii and iii are shown at higher magnification at right and highlight synaptic (i.e., utrophin and αBTX overlay) and extrasynaptic utrophin expression. Scale bars: 100 μm (low magnification) and 10 μm (insets). (E) Quantification of GAST muscle utrophin fluorescence intensity as a function of distance, showing synaptic and extrasynaptic expression in WT Veh, D2.mdx Veh and D2.mdx MK mice. Graphs display individual data points, group means (bars) and SEM. *n* = 8–10. Statistical significance is denoted as follows: *, *p* < 0.05 versus WT Veh; #, *p* < 0.05 versus D2.mdx Veh.

Our initial histological findings implicated an MK‐induced improvement in sarcolemma integrity, as previously described with other AMPK‐mediated adaptations in DMD muscle [11, 14, 15, 22]. To further investigate this observation, we examined the expression of key structural proteins, focusing on the dystrophin homologue, utrophin. Immunoblotting revealed a significant increase (+70%) in utrophin expression in the TA muscles of MK‐treated D2.mdx animals compared to Veh‐treated controls (Figure 6A,B). Similarly, the expression levels of utrophin‐associated proteins, including γ‐sarcoglycan (γSG) and β‐dystroglycan (βDG), were 10%–15% higher (*p* < 0.05) in MK‐treated animals than in Veh‐treated controls, approaching levels displayed in healthy WT mice (Figure 6A,B). Immunofluorescence labelling of γSG and βDG confirmed their sarcolemma localization in the GAST muscles, which was elevated after chronic AMPK activation (Figure 6C).

To assess fibre‐type‐specific patterns of utrophin, we conducted immunofluorescence microscopy experiments on serially cryosectioned GAST muscles, enabling differentiation between SO fibre‐enriched regions and predominantly FG areas (as shown in Figure 5A). We observed typical sarcolemma distribution of utrophin, including its localization at the periphery of SO muscle fibres and at the NMJ, along with compensatory upregulation in dystrophic muscle fibres (Figure 6D). In the SO regions of dystrophic GAST muscles, utrophin expression remained unchanged with MK treatment. However, an increased expression of synaptic and extrasynaptic utrophin was detected within FG regions of MK‐treated D2.mdx animals, indicating a fibre‐type‐specific adaptation (Figure 6D,E). Together, these observations show that utrophin upregulation accompanied the structural and functional adaptations elicited by MK‐induced AMPK stimulation in dystrophic skeletal muscle.

### 3.7 Daily AMPK Activation Restores Skeletal Muscle Mitochondrial Function and Reduces Mitochondrial Myopathy in D2.mdx Animals

**FIGURE 7:**
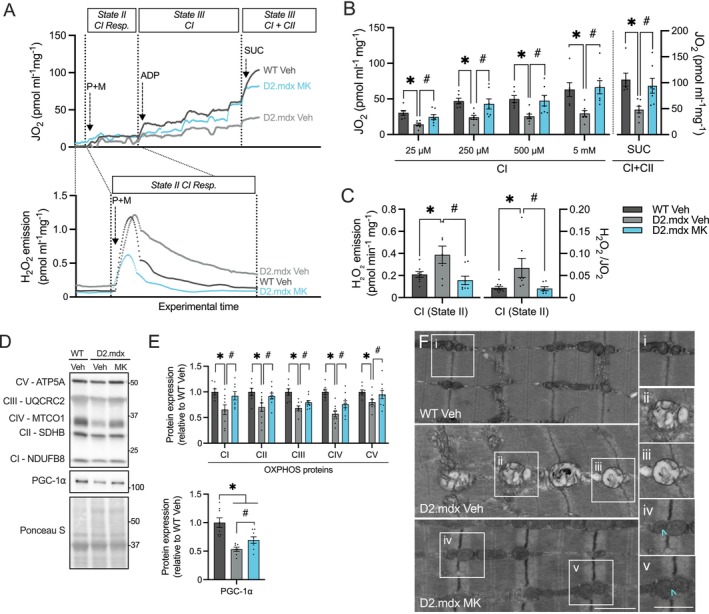
Daily AMPK activation restores skeletal muscle mitochondrial biology in D2.mdx animals. (A) Real‐time traces of simultaneous respiration (JO_2_; top) and H_2_O_2_ emission (bottom) of QUAD muscle fibre bundles from WT Veh, D2.mdx Veh and D2.mdx MK animals. Pyruvate (P), malate (M), ADP and succinate (SUC) substrate additions are indicated. Complex I (CI)‐linked State III respiration (JO_2_; pmol·mL^−1^·mg^−1^) at varying ADP concentrations (25 μM, 250 μM, 500 μM and 5 mM) and CI‐ and CII‐linked State III respiration (SUC). (B) Graphical summaries of CI‐linked and CI + CII‐linked State III respiration in QUAD muscles of Veh‐treated WT and Veh‐ or MK‐treated D2.mdx mice. (C) CI‐linked State II H_2_O_2_ emission (pmol·mL^−1^·mg^−1^) and free radical leak (H_2_O_2_/JO_2_) in QUAD muscles of Veh‐treated WT and Veh‐ or MK‐treated D2.mdx mice. Oxygen consumption and H_2_O_2_ emission values (pmol·mL^−1^) are expressed relative to the muscle fibre bundle wet weight (mg). (D) Representative western blots of mitochondrial electron transport chain (ETC) subunits [CI, NADH ubiquinone oxidoreductase subunit B8 (NDUFB8); CII, succinate dehydrogenase complex iron sulfur subunit B (SDHB); CIII, ubiquinol‐cytochrome c reductase core protein 2 (UQCRC2); CIV, mitochondrially encoded cytochrome c oxidase I (MTCO1); CV, mitochondrial α‐F_1_‐ATP synthase (ATP5A)] and peroxisome proliferator–activated receptor γ coactivator‐1α (PGC‐1α) in TA muscle lysates from Veh‐treated WT and Veh‐ or MK‐treated D2.mdx mice. Ponceau S staining is shown as a loading control. Protein ladder markers (kDa) are indicated. (E) Graphical summaries of protein levels related to mitochondrial ETC subunits involved in oxidative phosphorylation (OXPHOS) and PGC‐1α Veh‐treated WT and Veh‐ or MK‐treated D2.mdx mice. Data are presented as fold changes relative to the WT Veh group. (F) Representative electron microscopy images of intermyofibrillar mitochondria in the distal third of QUAD muscles from Veh‐ and MK‐treated mice. Insets show (i) healthy mitochondria in WT Veh muscle; (ii) swollen, dysmorphic mitochondria with onion‐shaped cristae; and (iii) dysmorphic mitochondria with linear cristae in D2.mdx Veh muscle; (iv, v) healthy organelles with mitochondrial contact sites in D2.mdx MK muscle. Cyan arrowheads indicate electron‐dense areas at mitochondrial membrane junctions. Scale bar: 1 μm. Graphs display individual data points, group means (bars) and SEM. *n* = 8–10. Statistical significance is denoted as follows: *, *p* < 0.05 versus WT Veh; #, *p* < 0.05 versus D2.mdx Veh.

We next aimed to determine whether AMPK‐mediated mitochondrial plasticity would extend to the application of MK in the more severe D2.mdx mouse model. Fibre bundles from the QUAD muscle were used for mitochondrial assessment. GAST and QUAD muscles show similar dystrophic severity in D2.mdx mice [31, 32]. High‐resolution respirometry revealed a 50%–55% decrease (*p* < 0.05) in complex I (CI)‐linked State III respiration, as well as a 55% reduction (*p* < 0.05) in CI + CII‐linked respiration, in QUAD muscles of Veh‐treated D2.mdx animals compared to healthy WT controls (Figure 7A,B). Remarkably, D2.mdx MK mice exhibited a significant elevation (+80%–125%) in CI‐linked and CI + CII State III respiration to levels similar to the WT Veh group (Figure 7B). Simultaneous measurement of reactive oxygen species (ROS) production revealed a 2‐fold increase (*p* < 0.05) in CI‐linked state II ROS production in Veh‐treated D2.mdx animals, which was attenuated (−70%; *p* < 0.05) with MK treatment (Figure 7C). Notably, these MK‐induced reductions persisted when ROS levels were normalized to oxygen flux, indicating a decrease in free radical leak.

As anticipated, the QUAD muscles of D2.mdx animals showed a significant decrease (20%–45%) in mitochondrial electron transport chain (ETC) complexes I–V (CI–CV) subunits when compared to healthy WT controls (Figure 7D,E). MK dosing increased (25%–45%; *p* < 0.05) the abundance of mitochondrial complexes CI, CII, CIII, CIV and CV, bringing their levels closer to those observed in healthy controls. Expression of peroxisome proliferator–activated receptor γ coactivator‐1α (PGC‐1α), a critical regulator of mitochondrial biogenesis, was 50% lower (*p* < 0.05) in the D2.mdx Veh group relative to their WT counterparts (Figure 7D,E). Following the MK intervention, PGC‐1α protein levels were 30% higher (*p* < 0.05) in the QUAD muscles of D2.mdx animals relative to their Veh‐treated controls. Markers of organelle fusion [i.e., optic atrophy 1 (OPA1), mitofusin 2 (MFN2), serine 637‐phosphorylated dynamin‐related protein 1 (pDRP^Ser637^)], fission [i.e., serine 616‐phosphorylated DRP1 (pDRP^Ser616^), fission protein 1 (FIS)] and mitophagy [i.e., PTEN‐induced kinase 1 (PINK)] were all differentially expressed between WT and dystrophic mice (Figure S4A,B), suggesting a collective imbalance favouring fission and a potential disruption in the clearance of damaged mitochondria in dystrophic muscle. MK did not impact these mitochondrial dynamics outcomes. Consistent with these data, electron microscopy revealed hallmarks of dysmorphic cellular organelles in the QUAD muscles of D2.mdx animals, including enlargement, swelling and altered cristae (Figure 7F). However, in the MK‐treated D2.mdx group, these abnormalities were markedly reduced. These observations collectively underscore the role of AMPK in mitigating mitochondrial myopathy in dystrophic muscle.

### 3.8 DMD Patient Muscle Exhibits a Mitochondrial Phenotype That Is Rescued Following Chronic AMPK Stimulation

Long‐term MK‐induced AMPK activation in D2.mdx mice led to multiple beneficial structural and functional adaptations, including improved skeletal muscle mitochondrial features. To assess translational relevance, we analysed gene expression from muscle biopsies of patients with DMD and Becker muscular dystrophy (BMD), a milder, genetically related form of DMD, compared to age‐ and sex‐matched healthy controls (GSE3307). Several mitochondrial ETC genes (e.g., *NDUFB8*, *SDHB*, *UQCRC3* and *COXII*) were downregulated in dystrophic patient muscle biopsies (Figure S5). This reduction in mitochondrial transcripts was more pronounced in DMD compared to BMD samples, implicating a correlation between disease severity and mitochondrial dysfunction.

**FIGURE 8:**
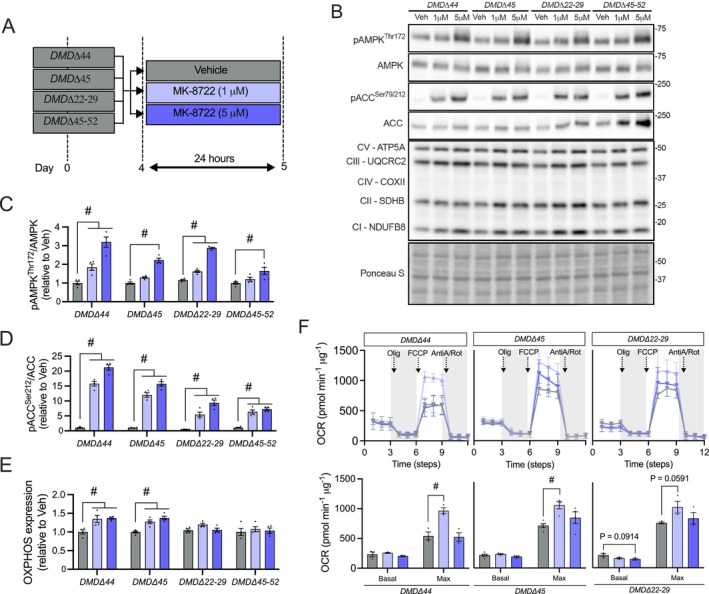
DMD patient‐derived skeletal muscle cells exhibit elevated mitochondrial content and respiration following chronic AMPK stimulation. (A) Schematic overview of in vitro Veh and MK dosing in patient‐derived cells. DMD patient‐derived myoblasts with different mutations (*DMDΔ44*, *DMDΔ45*, *DMDΔ22–29* and *DMDΔ45–52*) were cultured and differentiated into mature myotubes. After 4 days of differentiation, DMD lines were treated with Veh or MK (1 μM, 5 μM) for 24 h. (B) Representative Western blots showing pAMPK^Thr172^, AMPK, pACC^Ser212^, ACC and mitochondrial ETC subunits (CI‐V) in cell lysates from DMD patient‐derived lines. Ponceau S staining is shown as a loading control. Protein ladder markers are indicated in kDa. (C‐E) Graphical summaries of AMPK (C) and ACC (D) phosphorylation status, and total OXPHOS expression (i.e., sum of all mitochondrial ETC subunits expression) (E). Values are expressed relative to the Veh‐treated condition. (F) Real‐time oxygen consumption rate (OCR) traces during a mitochondrial stress test in Veh‐ and MK‐treated *DMDΔ44*, *DMDΔ45* and *DMDΔ22–29* cell lines. Graphical summaries of basal and maximal OCR in *DMDΔ44*, *DMDΔ45* and *DMDΔ22–29* muscle cells are shown below. OCR values are expressed relative to protein content (pmol·min^−1^·μg^−1^). Graphs display individual technical replicates, group means (bars) and SEM. *n* = 3–4. Statistical significance is denoted as follows: #, *p* < 0.05 versus Veh‐treated condition.

Lastly, we aimed to investigate whether these mitochondrial deficiencies in human DMD muscle could be ameliorated through repeated direct AMPK activation. Here, we administered MK (1 or 5 μM; 24 h) to DMD patient‐derived myotubes harbouring *DMDΔ44*, *DMDΔ45*, *DMDΔ22*–*29* and *DMDΔ45*–*52* mutations (Figure 8A) and then measured markers of organelle content and function. As anticipated, MK‐treated DMD myotubes exhibited significant increases in AMPK and ACC phosphorylation status across all DMD mutation lines (Figure 8B–D). Concomitantly, total OXPHOS expression (i.e., sum of all mitochondrial CI–CV marker levels) was elevated (+25%–35%; *p* < 0.05) in MK‐treated *DMDΔ44* and *DMDΔ45* myotubes when compared to their respective Veh‐treated controls (Figure 8B,E). No MK‐induced changes on OXPHOS were observed in *DMDΔ22*–*29* and *DMDΔ45*–*52* mutant lines. Our Seahorse XF analysis revealed that basal respiration was similar (*p* > 0.05) across all Veh‐ and MK‐treated DMD myotubes (Figure 8F). However, maximal respiration was significantly enhanced (+50%–80%) in MK‐treated *DMDΔ44* and *DMDΔ45* myotubes relative to Veh‐treated controls, with a trend toward elevated (+35%; *p* = 0.059) maximal respiration in *DMDΔ22*–*29* myotubes. Collectively, these DMD patient cell data recapitulate the beneficial effects of MK on mitochondrial biology in dystrophic animals and support our hypothesis that DMD patient muscle may undergo favourable adaptations in response to repeated AMPK activation.

## 4 Discussion

Our data demonstrate that long‐term, daily oral administration of a direct pharmacological AMPK activator effectively mitigates physiological and molecular hallmarks of DMD in severe D2.mdx mice. Furthermore, we observed improved mitochondrial biology in DMD patient‐derived muscle cells with MK dosing, an adaptation that addresses a well‐documented skeletal muscle mitochondrial respiratory deficit in patients [8]. Collectively, our findings support several earlier studies demonstrating the safety and effectiveness of chronic AMPK activation in attenuating the progression of DMD [8, 9, 11, 15, 22], as well as other NMDs [8, 9, 21], and identify direct, small molecule AMPK agonists as a potentially promising new class of DMD mutation‐agnostic therapies.

AMPK is an ancient, highly conserved enzyme that governs a wide array of cellular processes [7, 20], several of which are disrupted in the pathophysiology of DMD [1, 8, 9]. To explore the convergence of AMPK activity with dystrophic molecular pathology, we integrated pre‐existing transcriptomic datasets from DMD patient skeletal muscle samples (GSE38417) with results of pharmacological (i.e., MK) and physiological (i.e., exercise) AMPK stimulation in mice [GSE92719 [24]]. This analysis revealed that DMD‐associated transcriptional patterns linked to aberrant ECM remodelling, or fibrosis, as well as dysregulated mitochondrial function, were attenuated by these modes of AMPK stimulation. These findings were consistent with our data from a single dose of MK in D2.mdx mice, as well as with results obtained from MK dosing in the traditional mdx model [22]. Moreover, they align with several previous studies that have investigated the modulation of individual cellular pathways by AMPK activation in dystrophic skeletal muscle, with a particular emphasis on autophagy [14, 33, 34], mitochondrial biogenesis [11, 13, 18, 22, 35, 36, 37] and inflammation resolution [16, 17]. However, species‐specific regulatory differences and the short duration of MK treatment provided limited insight into long‐term functional outcomes. This motivated us to test whether sustained AMPK activation via chronic MK dosing could produce durable physiological and histopathological benefits and offer a mutation‐independent therapeutic strategy for DMD.

MK is a direct, potent and orally bioavailable AMPK agonist capable of sustained target engagement across multiple tissues [20]. Our findings extend the application of this type of AMPK activation to the DMD context and assert that repeated MK dosing improves muscle performance in vivo and ex vivo by enhancing muscular endurance, exercise tolerance and contractile force all while maintaining muscle mass and myofibre size. These functional gains suggest that the benefits of AMPK activation arise, at least in part, from the demonstrated improvements in metabolic function and structural resilience, rather than hypertrophy or regenerative mechanisms. Although serum creatine kinase could not be measured in the current study, the reductions in IgG infiltration and fibrosis observed with MK treatment strongly suggest improved sarcolemmal stability. Indeed, we observed coincident increases in mitochondrial capacity, NMJ integrity and membrane‐stabilizing proteins including utrophin and DAPC components in the MK‐treated cohort. These findings are consistent with a recent report in a murine model of myotonic dystrophy Type I treated with MK, in which the animals demonstrated a shift towards a slower, more oxidative muscle phenotype [21]. Additionally, our data build upon previous observations of AMPK‐induced utrophin expression by identifying a fibre‐type‐specific upregulation pattern, with prominent increases in fast glycolytic fibres [11, 37]. This may reflect the capacity of MK to promote oxidative remodelling in muscle subsets most vulnerable to degeneration.

We recently demonstrated that endurance‐type voluntary exercise improves muscle function, reduces fibrosis and enhances mitochondrial biology in D2.mdx animals without exacerbating dystrophic pathology [38]. Thus, like the effects of long‐term volitional exercise, our current findings demonstrate that chronic MK treatment did not cause overt hepatic steatosis (data not shown) or exacerbate cardiac pathology in D2.mdx mice. Given that we found comparable physiological and transcriptional signatures between MK treatment and regular exercise, these data suggest that MK may mimic key aspects of endurance exercise. This is particularly relevant in the context of DMD, where not all patients are able to engage in sufficient physical activity to obtain such benefits, highlighting MK as a potentially viable pharmacological alternative. Additionally, echocardiographic analyses confirmed the similarity in heart structure and function between Veh‐ and MK‐treated dystrophic animals and in fact showed that mitral valve function returned to healthy, WT levels. These observations are particularly important given the progressive nature of cardiac pathology in DMD and are highly relevant considering prior reports describing MK‐induced, reversible cardiac hypertrophy with glycogen accumulation in both mice and non‐human primates [25, 28]. Notably, reversible cardiac remodelling and glycogen accumulation are also observed with chronic endurance exercise [39]. Nevertheless, the development of next‐generation MK‐related agents with shorter half‐lives (‘short‐acting’ variants) may offer a more favourable therapeutic option [24]. Furthermore, the development of next‐generation AMPK activators that selectively engage skeletal muscle‐relevant isoform compositions (i.e., α2β2γ1 or α2β2γ3) may offer a more precise and effective approach. The application of such compounds in DMD muscle represents a promising avenue for further investigation. Importantly, longer‐term longitudinal studies, including those conducted in animals at more advanced stages of disease, will also be essential for evaluating the durability of therapeutic effects and identifying any potential delayed adverse outcomes associated with prolonged AMPK activation.

Targeting modifiable cellular processes in muscular dystrophy, such as mitochondrial dysfunction or chronic inflammation, represents a compelling strategy for developing mutation‐independent therapies [40]. Our findings reinforce this paradigm and corroborate previous reports [8] by highlighting a consistent mitochondrial deficit across D2.mdx mice, DMD patient biopsies and cultured patient‐derived myotubes. MK ameliorated this phenotype by restoring mitochondrial respiration and attenuating ROS production, enhancing the expression of ETC proteins and improving organelle ultrastructure in the skeletal muscle of D2.mdx mice. These data are consistent with evidence demonstrating similar mitochondrial improvements from proof‐of‐concept studies employing indirect AMPK activators in preclinical DMD models [9, 11, 18, 35, 41], as well as with enhanced muscle function outcomes from a clinical trial of MET in DMD patients [42]. Furthermore, these beneficial effects translated to the human DMD condition as direct AMPK stimulation with MK increased OXPHOS protein expression and elevated maximal mitochondrial respiration in DMD patient‐derived myotubes harbouring a variety of mutations within the *DMD* gene, including those located at established mutation hotspots. This mutation‐independent amelioration is particularly compelling, as it suggests that long term, direct AMPK activation may exhibit therapeutic efficacy across all DMD patients. Combination therapies leveraging both targeted dystrophin‐dependent strategies such as exon skipping, microgene replacement and nonsense suppression [3, 4], with dystrophin‐independent approaches capitalizing on the effects of AMPK, including enhanced mitochondrial biology, may attenuate the broad pathophysiology of DMD.

In conclusion, our findings delineate direct AMPK activation as a promising therapeutic strategy in the context of DMD, with MK serving as an orally bioavailable and robust representative pharmacological tool to demonstrate this effect. The application of MK in the present study underscores the broader utility of sustained and systemic direct AMPK engagement in conferring metabolic and structural benefits to dystrophic muscle, without exacerbating deleterious cardiac phenotypes. Ultimately, our findings provide strong preclinical evidence for the continued development of modalities directly targeting AMPK and build on an abundance of previous work [9, 11, 14, 15, 18, 21, 22, 35, 41, 42] that supports the use of AMPK activators as potential mutation‐independent therapies with clinical relevance for all individuals affected by DMD.

## Funding

This work was funded by the Canadian Institutes of Health Research (Project Grant: 408289 to V.L.), the Canada Research Chairs program (Tier 2: 232338 to V.L.) and the Ontario Ministry of Economic Development, Job Creation and Trade (Early Researcher Award: ER17‐13‐087 to V.L.). During the time of this study, S.Y.N., S.R.M. and S.I.H. were supported by the Natural Sciences and Engineering Research Council of Canada Scholarships. A.I.M. was supported by an Ontario Graduate Scholarship.

## Conflicts of Interest

G.R.S. is a cofounder and shareholder of Espervita Therapeutics, a company developing new medications for fibrosis and cancer. McMaster University has received funding from Cambrian Biosciences, Catalym, Espervita Therapeutics, Esperion Therapeutics, Merck, Nestle, Novo Nordisk and Poxel Pharmaceuticals for research conducted in the laboratory of G.R.S. G.R.S. has received consulting and speaking fees from AstraZeneca, CurieBio, Eli Lilly, Esperion Therapeutics, Korro Bio, Keros Therapeutics, Merck, Novo Nordisk, Versant Ventures and Poxel Pharmaceuticals. M.A.T. is the founder, CEO and CSO of Exerkine Corporation and Stayabove Nutrition, a biotechnology company that develops and commercializes therapies based on nutritional supplements (Stayabove Nutrition), exercise‐derived factors (‘exerkines’) to treat and diagnose genetic disorders and chronic diseases.

## Supporting information


**Data S1:** Supporting Information.

**Table S1:** Patient‐derived myotube characteristics.
**Figure S1:** Chronic MK treatment does not exacerbate the dystrophic cardiac phenotype in D2.mdx animals. (A) Representative M‐mode in vivo echocardiography images in WT Veh, D2.mdx Veh, and D2.mdx MK mice during Week 7 of Veh or MK treatment. (B) Summary of predicted cardiac morphology metrics (millimetres; mm), including left ventricle anterior wall thickness (LVAW), left ventricle internal diameter (LVID) and left ventricle posterior wall thickness (LVPW) during systole and diastole. (C) Heart mass relative to body mass. (D) Relative ventricular septum wall thickness during systole and diastole in WT Veh, D2.mdx Veh, and D2.mdx MK mice. (E) Fractional shortening (FS) and ejection fraction (EF) expressed as percentages. (F) Cardiac output (mL min^−1^) in WT Veh‐ and Veh‐ and MK‐treated D2.mdx animals. (G) Representative mitral valve E and A wave images in WT Veh, D2.mdx Veh and D2.mdx MK animals. (H–N) Summaries of mitral valve metrics, including peak velocity (H) and deceleration (I) of E waves, peak velocity (J) and acceleration (K) of A waves, E/A peak velocity ratio (L), as well as isovolumetric (IV) contraction and relaxation times (M), and myocardial performance index (N). Graphical summaries show individual data points and group means with SEM (*n* = 5–6). (O–P) Sirus Red and haematoxylin and eosin staining on cardiac sections from WT Veh, D2.mdx Veh and D2.mdx MK mice. Scale bar: 100 μm. (Q) Quantification of cardiac fibrosis area (%) in WT Veh, D2.mdx Veh and D2.mdx MK animals. Statistical significance indicated by #, *p* < 0.05 versus Veh‐treated D2.mdx animals.
**Figure S2:** Repeated MK treatment enlarges postsynaptic features of the neuromuscular junction (NMJ) in skeletal muscles of D2.mdx animals. (A) Confocal IF microscopy images of NMJs in the epitrochleoanconeus muscle of Veh‐treated WT and Veh‐ or MK‐treated D2.mdx animals. Presynaptic morphology is visualized using labels for neurofilament M and synaptic vesicle 2 (NFM + SV2, magenta), and AChRs at the motor endplate are presented using αBTX (green). Scale bar: 20 μm. (B‐C) Pre‐ and postsynaptic NMJ morphology metrics for all experimental groups, derived from NMJmorph analysis. Graphical summaries show individual data points and group means with SEM (*n* = 4–5). Statistical significance indicated by #, *p* < 0.05 versus Veh‐treated D2.mdx animals.
**Figure S3:** Skeletal muscle mass and myofibre morphology analyses in D2.mdx mice following MK treatment. (A) Muscle mass of quadriceps (QUAD), gastrocnemius (GAST), TA and triceps (TRI) from Veh‐ and MK‐treated animals. Values are expressed relative to body mass (mg g^−1^). (B) Circle graphs of myosin heavy chain fibre type distributions in GAST muscles from WT Veh, D2.mdx Veh, and D2.mdx MK mice. (C,D) Graphical summaries of average minimum Feret's diameter (μm) (C) and fibre size variation (expressed as coefficient of variation percentage) (D) in the slow oxidative and fast glycolytic regions of GAST muscles. Data are presented as fold changes relative to the WT Veh group. Graphs display individual data points, group means (bars) and SEM. *n* = 8–10. Statistical significance is denoted as follows: *, *p* < 0.05 versus WT Veh; #, *p* < 0.05 versus D2.mdx Veh.
**Figure S4:** Mitochondrial dynamic proteins are disrupted in D2.mdx muscle but unaffected by MK treatment. (A) Representative western blots of optic atrophy 1 (OPA1), mitofusin‐2 (MFN2), serine (Ser) 637‐phosphorylated DRP (pDRP^Ser637^), Ser616‐phosphorylated dynamin‐related protein (pDRP^Ser616^), DRP, fission protein 1 (FIS1) and PTEN‐induced kinase 1 (PINK) in TA muscle lysates from Veh‐treated WT and Veh‐ or MK‐treated D2.mdx mice. Ponceau S staining is shown as a loading control. Protein ladder markers (kDa) are indicated. (B) Graphical summaries of protein levels related to mitochondrial fusion, fission and mitophagy in Veh‐treated WT and Veh‐ or MK‐treated D2.mdx mice. Data are presented as fold changes relative to the WT Veh group. Graphs display individual data points, group means (bars) and SEM. *n* = 8–10. Statistical significance is denoted as: *, *p* < 0.05 versus WT Veh; #, *p* < 0.05 versus D2.mdx Veh.
**Figure S5:** DMD patient skeletal muscle biopsies show downregulation of mitochondrial complex I genes. Heat map showing expression of mitochondrial ETC genes curated from the Gene Ontology (GO) category ‘Mitochondrial electron transport, NADH to ubquinone; succinate to ubiquinone; cytochrome c to oxygen, ubiquinol to cytochrome c’ (GO:0006120; GO: 0006121; GO: 0006123; GO: 0006122) in skeletal muscle biopsies from healthy controls, Becker muscular dystrophy (BMD) and DMD patients from the GSE3307 dataset.

## Data Availability

All data supporting the findings of this study are included in the manuscript and are available from the corresponding author upon reasonable request.

## References

[1] D. Duan , N. Goemans , S. Takeda , E. Mercuri , and A. Aartsma‐Rus , “Duchenne Muscular Dystrophy,” Nature Reviews Disease Primers 7 (2021): 1–19. 10.1038/s41572-021-00248-3 PMC10557455 33602943

[2] A. E. H. Emery , “Population Frequencies of Inherited Neuromuscular Diseases‐A World Survey,” Neuromuscular Disorders 1 (1991): 19–29. 1822774 10.1016/0960-8966(91)90039-u

[3] N. E. Bengtsson , H. Tasfaout , and J. S. Chamberlain , “The Road Toward AAV‐Mediated Gene Therapy of Duchenne Muscular Dystrophy,” Molecular Therapy 33 (2025): 2035–2051. 40181545 10.1016/j.ymthe.2025.03.065 PMC12126791

[4] A. Aartsma‐Rus , “The Future of Exon Skipping for Duchenne Muscular Dystrophy,” Human Gene Therapy 34 (2023): 372–378. 36924282 10.1089/hum.2023.026

[5] H. Wilton‐Clark and T. Yokota , “Biological and Genetic Therapies for the Treatment of Duchenne Muscular Dystrophy,” Expert Opinion on Biological Therapy 23 (2023): 49–59. 36409820 10.1080/14712598.2022.2150543

[6] G. R. Steinberg and B. E. Kemp , “AMPK in Health and Disease,” Physiological Reviews 89 (2009): 1025–1078. 19584320 10.1152/physrev.00011.2008

[7] G. R. Steinberg and D. G. Hardie , “New Insights Into Activation and Function of the AMPK,” Nature Reviews. Molecular Cell Biology 24 (2023): 255–272. 36316383 10.1038/s41580-022-00547-x

[8] A. I. Mikhail , S. Y. Ng , S. R. Mattina , and V. Ljubicic , “AMPK Is Mitochondrial Medicine for Neuromuscular Disorders,” Trends in Molecular Medicine 29 (2023): 512–529. 37080889 10.1016/j.molmed.2023.03.008

[9] A. G. Dial , S. Y. Ng , A. Manta , and V. Ljubicic , “The Role of AMPK in Neuromuscular Biology and Disease,” Trends in Endocrinology and Metabolism 29 (2018): 300–312. 29572064 10.1016/j.tem.2018.02.010

[10] S. Y. Ng , A. I. Mikhail , S. R. Mattina , et al., “AMPK Regulates the Maintenance and Remodelling of the Neuromuscular Junction,” Molecular Metabolism 91 (2025): 102066. 39571900 10.1016/j.molmet.2024.102066 PMC11646796

[11] V. Ljubicic , P. Miura , M. Burt , et al., “Chronic AMPK Activation Evokes the Slow, Oxidative Myogenic Program and Triggers Beneficial Adaptations in mdx Mouse Skeletal Muscle,” Human Molecular Genetics 20 (2011): 3478–3493. 21659335 10.1093/hmg/ddr265

[12] V. Ljubicic , M. Burt , and B. J. Jasmin , “The Therapeutic Potential of Skeletal Muscle Plasticity in Duchenne Muscular Dystrophy: Phenotypic Modifiers as Pharmacologic Targets,” FASEB Journal 28 (2014): 548–568. 24249639 10.1096/fj.13-238071

[13] C. Péladeau , A. Ahmed , A. Amirouche , et al., “Combinatorial Therapeutic Activation With Heparin and AICAR Stimulates Additive Effects on Utrophin A Expression in Dystrophic Muscles,” Human Molecular Genetics 25 (2016): 24–43. 26494902 10.1093/hmg/ddv444 PMC4690489

[14] M. Pauly , F. Daussin , Y. Burelle , et al., “AMPK Activation Stimulates Autophagy and Ameliorates Muscular Dystrophy in the mdx Mouse Diaphragm,” American Journal of Pathology 181 (2012): 583–592. 22683340 10.1016/j.ajpath.2012.04.004

[15] X. Dong , T. Hui , J. Chen , et al., “Metformin Increases Sarcolemma Integrity and Ameliorates Neuromuscular Deficits in a Murine Model of Duchenne Muscular Dystrophy,” Frontiers in Physiology 12 (2021): 606. 10.3389/fphys.2021.642908 PMC8126699 34012406

[16] G. Juban , M. Saclier , H. Yacoub‐Youssef , et al., “AMPK Activation Regulates LTBP4‐Dependent TGF‐β1 Secretion by Pro‐Inflammatory Macrophages and Controls Fibrosis in Duchenne Muscular Dystrophy,” Cell Reports 25 (2018): 2163–2176.e6. 30463013 10.1016/j.celrep.2018.10.077

[17] B. S. Gordon , D. C. Delgado Díaz , and M. C. Kostek , “Resveratrol Decreases Inflammation and Increases Utrophin Gene Expression in the mdx Mouse Model of Duchenne Muscular Dystrophy,” Clinical Nutrition 32 (2013): 104–111. 22795790 10.1016/j.clnu.2012.06.003

[18] H. Al‐Rewashdy , V. Ljubicic , W. Lin , J. M. Renaud , and B. J. Jasmin , “Utrophin A Is Essential in Mediating the Functional Adaptations of mdx Mouse Muscle Following Chronic AMPK Activation,” Human Molecular Genetics 24 (2015): 1243–1255. 25324540 10.1093/hmg/ddu535

[19] A. Kuno , R. Hosoda , R. Sebori , et al., “Resveratrol Ameliorates Mitophagy Disturbance and Improves Cardiac Pathophysiology of Dystrophin‐Deficient mdx Mice,” Scientific Reports 8 (2018): 15555. 30348945 10.1038/s41598-018-33930-w PMC6197260

[20] G. R. Steinberg and D. Carling , “AMP‐Activated Protein Kinase: The Current Landscape for Drug Development,” Nature Reviews Drug Discovery 18, no. 7 (2019): 527–551. 30867601 10.1038/s41573-019-0019-2

[21] A. Ravel‐Chapuis , C. Fahmi , J. Gobin , and B. J. Jasmin , “The AMPK Allosteric Activator MK‐8722 Improves the Histology and Spliceopathy in Myotonic Dystrophy Type 1 (DM1) Skeletal Muscle,” FASEB Journal 38 (2024): e70199. 39611312 10.1096/fj.202401145RR

[22] S. Y. Ng , A. I. Mikhail , S. R. Mattina , A. Manta , I. J. Diffey , and V. Ljubicic , “Acute, Next‐Generation AMPK Activation Initiates a Disease‐Resistant Gene Expression Program in Dystrophic Skeletal Muscle,” FASEB Journal 37 (2023): e22863. 37016990 10.1096/fj.202201846RR

[23] S. D. Lombardo , E. Mazzon , K. Mangano , et al., “Transcriptomic Analysis Reveals Involvement of the Macrophage Migration Inhibitory Factor Gene Network in Duchenne Muscular Dystrophy,” Genes 10 (2019): 939. 31752120 10.3390/genes10110939 PMC6896047

[24] E. S. Muise , H.‐P. Guan , J. Liu , et al., “Pharmacological AMPK Activation Induces Transcriptional Responses Congruent to Exercise in Skeletal and Cardiac Muscle, Adipose Tissues and Liver,” PLoS ONE 14 (2019): e0211568. 30811418 10.1371/journal.pone.0211568 PMC6392219

[25] R. W. Myers , H.‐P. Guan , J. Ehrhart , et al., “Systemic Pan‐AMPK Activator MK‐8722 Improves Glucose Homeostasis but Induces Cardiac Hypertrophy,” Science 357 (2017): 507–511. 28705990 10.1126/science.aah5582

[26] M. Calore , “The PRKAG2 Gene and Hypertrophic Cardiomyopathy: An Energetically Imbalanced Relationship,” American Journal of Physiology. Heart and Circulatory Physiology 313 (2017): H248–H250. 28626079 10.1152/ajpheart.00316.2017

[27] D. J. Birnkrant , K. Bushby , C. M. Bann , et al., “Diagnosis and Management of Duchenne Muscular Dystrophy, Part 1: Diagnosis, and Neuromuscular, Rehabilitation, Endocrine, and Gastrointestinal and Nutritional Management,” Lancet Neurology 17 (2018): 251–267. 29395989 10.1016/S1474-4422(18)30024-3 PMC5869704

[28] D. J. Birnkrant , L. Bello , R. J. Butterfield , et al., “Cardiorespiratory Management of Duchenne Muscular Dystrophy: Emerging Therapies, Neuromuscular Genetics, and New Clinical Challenges,” Lancet Respiratory Medicine 10 (2022): 403–420. 35364035 10.1016/S2213-2600(21)00581-6

[29] D. W. Hammers , C. C. Hart , M. K. Matheny , et al., “The D2.mdx Mouse as a Preclinical Model of the Skeletal Muscle Pathology Associated With Duchenne Muscular Dystrophy,” Scientific Reports 10 (2020): 1–12. 32826942 10.1038/s41598-020-70987-y PMC7442653

[30] S. Y. Ng and V. Ljubicic , “Recent Insights Into Neuromuscular Junction Biology in Duchenne Muscular Dystrophy: Impacts, Challenges, and Opportunities,” eBioMedicine 61 (2020): 103032. 33039707 10.1016/j.ebiom.2020.103032 PMC7648118

[31] W. D. Coley , L. Bogdanik , M. C. Vila , et al., “Effect of Genetic Background on the Dystrophic Phenotype in MDX Mice,” Human Molecular Genetics 25, no. 1 (2016): 130–145. 26566673 10.1093/hmg/ddv460 PMC4690497

[32] M. van Putten , K. Putker , M. Overzier , et al., “Natural Disease History of the D2‐MDX Mouse Model for Duchenne Muscular Dystrophy,” FASEB Journal: Official Publication of the Federation of American Societies for Experimental Biology 33, no. 7 (2019): 8110–8124. 30933664 10.1096/fj.201802488R PMC6593893

[33] C. De Palma , F. Morisi , S. Cheli , et al., “Autophagy as a New Therapeutic Target in Duchenne Muscular Dystrophy,” Cell Death & Disease 5 (2014): e1363. 25101676 10.1038/cddis.2014.312 PMC4454298

[34] E. Fiacco , F. Castagnetti , V. Bianconi , et al., “Autophagy Regulates Satellite Cell Ability to Regenerate Normal and Dystrophic Muscles,” Cell Death and Differentiation 23 (2016): 1839–1849. 27447110 10.1038/cdd.2016.70 PMC5071573

[35] V. Ljubicic and B. J. Jasmin , “Metformin Increases Peroxisome Proliferator‐Activated Receptor γ Co‐Activator‐1α and Utrophin A Expression in Dystrophic Skeletal Muscle,” Muscle & Nerve 52 (2015): 139–142. 25908446 10.1002/mus.24692

[36] V. Ljubicic , M. Burt , J. A. Lunde , and B. J. Jasmin , “Resveratrol Induces Expression of the Slow, Oxidative Phenotype in mdx Mouse Muscle Together With Enhanced Activity of the SIRT1‐PGC‐1 Axis,” American Journal of Physiology‐Cell Physiology 307 (2014): C66–C82. 24760981 10.1152/ajpcell.00357.2013 PMC4080183

[37] V. Ljubicic , S. Khogali , J.‐M. Renaud , and B. J. Jasmin , “Chronic AMPK Stimulation Attenuates Adaptive Signaling in Dystrophic Skeletal Muscle,” American Journal of Physiology‐Cell Physiology 302 (2012): C110–C121. 21940670 10.1152/ajpcell.00183.2011

[38] S. R. Mattina , S. Y. Ng , A. I. Mikhail , et al., “Volitional Exercise Elicits Physiological and Molecular Improvements in the Severe D2.mdx Mouse Model of Duchenne Muscular Dystrophy,” Journal of Physiology 604 (2025): 849–867. 40189894 10.1113/JP286768 PMC12810227

[39] B. C. Bernardo , K. L. Weeks , L. Pretorius , and J. R. McMullen , “Molecular Distinction Between Physiological and Pathological Cardiac Hypertrophy: Experimental Findings and Therapeutic Strategies,” Pharmacology & Therapeutics 128 (2010): 191–227. 20438756 10.1016/j.pharmthera.2010.04.005

[40] C. A. Timpani , A. Hayes , and E. Rybalka , “Revisiting the Dystrophin‐ATP Connection: How Half a Century of Research Still Implicates Mitochondrial Dysfunction in Duchenne Muscular Dystrophy Aetiology,” Medical Hypotheses 85 (2015): 1021–1033. 26365249 10.1016/j.mehy.2015.08.015

[41] V. Ljubicic and B. J. Jasmin , “AMP‐Activated Protein Kinase at the Nexus of Therapeutic Skeletal Muscle Plasticity in Duchenne Muscular Dystrophy,” Trends in Molecular Medicine 19 (2013): 614–624. 23891277 10.1016/j.molmed.2013.07.002

[42] P. Hafner , U. Bonati , A. Klein , et al., “Effect of Combination l‐Citrulline and Metformin Treatment on Motor Function in Patients With Duchenne Muscular Dystrophy: A Randomized Clinical Trial,” JAMA Network Open 2 (2019): e1914171. 31664444 10.1001/jamanetworkopen.2019.14171 PMC6824222

