# Supplementary material for: Direct AMPK Activation Confers Mutation‐Independent Therapeutic Benefit in Duchenne Muscular Dystrophy
Source: J Cachexia Sarcopenia Muscle. 2026 Feb 4;17(1):e70200. doi: 10.1002/jcsm.70200 (PMC12872333; doi:10.1002/jcsm.70200)
Supplement: Supplementary file 1 — Data S1: Supporting Information. [file JCSM-17-e70200-s002.docx]

**SUPPLEMENTARY MATERIALS AND METHODS**

**Animal experiments**

Male DBA/2J wild-type (WT, JAX #000671) and DBA/2J-mdx (D2.mdx; JAX #013141) mice were obtained from The Jackson Laboratory. D2.mdx mice are dystrophin-deficient and harbor a polymorphism in the *Ltbp4* gene, a known genetic modifier of Duchenne muscular dystrophy severity ^1,2^. Animals were housed in a pathogen-free facility on a 12-hour light/dark cycle with ad libitum access to water and standard chow. All experiments were performed in accordance with institutional animal care and use guidelines.

Five-week-old male D2.mdx mice received a single oral gavage of MK (5 mg/kg; provided via Material Transfer Agreement with Merck & Co.). This dose achieves sustained AMPK activation sufficient to improve metabolic and mitochondrial function while remaining below levels (>10 mg/kg) linked to reversible cardiac hypertrophy ^3^. MK was formulated in vehicle solution (0.25% methylcellulose, 5% polysorbate 80, 0.02% sodium lauryl sulfate). Mice were euthanized by cervical dislocation at 1, 3, or 12 hours post-gavage. Vehicle-treated mice were euthanized at matching timepoints and pooled to form a single control group. Tibialis anterior (TA) muscles were dissected and immediately flash frozen in liquid nitrogen.

Additional groups of five-week-old male D2.mdx mice were treated daily via oral gavage for 7 weeks with either vehicle (as above) or MK (5 mg/kg). WT mice treated with vehicle served as healthy controls. All treatments were administered between 0900–1200 hours daily. After 6 weeks of treatment, mice underwent in vivo muscle strength testing, metabolic cage monitoring, and echocardiographic assessment. At the end of week 7, animals were euthanized, and skeletal muscles were harvested for ex vivo muscle force measurement and high-resolution respirometry. Additional tissues were dissected, weighed, embedded in OCT compound, fixed for electron microscopy, or flash frozen in liquid nitrogen for downstream analyses.

**Cell experiments**

Human myoblasts were derived from skeletal muscle biopsies obtained from DMD patients at Fondazione IRCCS, Istituto Neurologico Carlo Besta. Patient characteristics are summarized in Table 1. Cells were maintained in growth medium consisting of Ham’s F10 nutrient mix (Thermo Fisher Scientific, 11550043) supplemented with 20% fetal bovine serum (Thermo Fisher Scientific, 12483020), dexamethasone (0.4 µg/mL; Sigma-Aldrich, D4902), fibroblast growth factor (10 µg/mL; Life Technologies, PHG0264), insulin (5 µg/mL; DIN 00586714), and 1% penicillin-streptomycin (Thermo Fisher Scientific, 15140122). All cultures were maintained at 37°C in a humidified incubator with 5% CO₂. Myogenic differentiation was initiated once cultures reached full confluence by replacing the growth medium with differentiation medium composed of Ham’s F10, 2% horse serum, and 1% penicillin-streptomycin. All in vitro experiments were performed using cells between passages 3 and 7 to ensure consistency and minimize variability due to passaging. Feeding and collection time points were standardized across experiments.

For pharmacological experiments, differentiated myotubes were treated for 24 hours with either vehicle (0.01% DMSO; Sigma-Aldrich, D2650) or MK (1 µM or 5 µM; provided via Material Transfer Agreement with Merck & Co.). The 1 and 5 μM MK concentrations were chosen based on our previous work^4^, showing that 2.5–10 μM MK robustly activates AMPK signaling. At the conclusion of the treatment period, cells were either harvested for protein extraction or subjected to Seahorse XF metabolic flux analysis.

**Animal Phenotyping and functional testing**

*Body Composition Assessment*

Body weight and body composition were evaluated following chronic vehicle or MK treatment. Fat and lean mass were quantified using a Minispec Whole Body Composition Analyzer (Bruker, Billerica, MA, USA). Body composition values were expressed as both absolute mass (g) and percentage of body weight, calculated as [(fat or lean mass in g) / body weight in g] × 100.

*Whole-Body Metabolism and Ambulatory Behavior*

Metabolic phenotyping was performed using the Promethion metabolic cage system (Sable Systems International, North Las Vegas, NV, USA). Mice were allowed to acclimatize for 12 hours prior to data acquisition. Parameters including oxygen consumption (VO₂), carbon dioxide production (VCO₂), energy expenditure, respiratory exchange ratio (RER), food intake, and cage activity were recorded every 5 minutes. Fatty acid oxidation was calculated using the equation: (1.70 × VO₂) − (1.69 × VCO₂), expressed in mg·kg⁻¹·h⁻¹.

Ambulatory activity was measured using the Opto-Varimex-5 Auto-Track open-field test (Columbus Instruments, Columbus, OH, USA), following the Treat-NMD protocol DMD_M.2.1.002. Mice were habituated to the arena for 5 minutes the day prior to testing. On test day, each animal was placed in the center of the open field and allowed to explore for 10 minutes. Behavioral data were collected and analyzed using Auto-Track software.

*In Vivo Muscle Function Testing*

Muscle strength and fatigue were assessed using a grid-grip dynamometer (Columbus Instruments), adapted from Treat-NMD SOP DMD_M.2.2.001. Each mouse performed seven sets of three consecutive pull attempts, with 2-minute rest intervals between sets. The three highest values were averaged to determine maximal grip strength, and relative strength was calculated by normalizing to body weight. Grip fatigue was calculated as [(maximum − minimum force) / maximum force] × 100.

Four-limb hanging performance was tested using a suspended wire grid in accordance with Treat-NMD SOP DMD_M.2.1.005. Mice underwent three trials (5 minutes rest between trials), and total hold time was multiplied by body weight to calculate holding impulse.

Exercise endurance was assessed via a treadmill-based exhaustion protocol (Treat-NMD SOP DMD_M.2.1.003). Mice were habituated to treadmill running for five days. The exhaustion test was performed one week prior to tissue collection to avoid acute exercise effects. Mice began running at 5 m·min⁻¹ with a gradual increase of 1 m·min⁻². Exhaustion was defined as the inability to continue running for 10 seconds despite repeated nudges. All assessments were conducted by a blinded investigator.

*Ex Vivo Muscle Contractility*

Contractile function of the extensor digitorum longus (EDL) muscle was evaluated using an Aurora Scientific 1300A system (Aurora, ON, Canada). EDL muscles were dissected and mounted in oxygenated Ringer’s solution (120 mM NaCl, 4.7 mM KCl, 2.5 mM CaCl₂, 1.2 mM KH₂PO₄, 1.2 mM MgSO₄, 25 mM HEPES, 5.5 mM glucose). After 10 minutes of equilibration, muscles were stimulated to determine optimal length and voltage based on previously described methods ^5^. Twitch contractions were used to determine peak force, time to peak tension, rates of force development and relaxation (+dF/dt and −dF/dt), and half relaxation time.

A force-frequency curve was generated by stimulating the muscle at increasing frequencies (10–140 Hz) at 30-second intervals. To assess eccentric contraction-induced damage, 10 lengthening contractions were administered every 2 minutes using supramaximal stimulation (700 ms train, 200 Hz, with 10% stretch during final 200 ms at 0.5 Le/s). Isometric force immediately before and after each contraction was used to calculate relative force drop. Data acquisition and analysis were performed using Dynamic Muscle Control Software (version 615A, Aurora Scientific). EDL muscles were subsequently embedded in OCT and frozen in isopentane for further analysis.

*Echocardiographic Assessment*

Transthoracic echocardiography was performed using the Prospect T1 system (Scintica, ON, Canada) four to five days before tissue harvest as previously described ^6^. Mice were anesthetized with 3% isoflurane in oxygen, and heart rates were maintained between 350–450 bpm. Animals were placed on a heated imaging platform to maintain normothermia (37°C).

M-mode echocardiographic images of the left ventricle (LV) short axis were acquired at the papillary muscle level to evaluate systolic function, including ejection fraction (EF), stroke volume (SV), fractional shortening (FS), left ventricular mass, and end-diastolic volume (EDV). FS was calculated as [(LVDd − LVDs)/LVDd] × 100, and EF as SV/EDV. Cardiac output was estimated as SV × heart rate.

Diastolic function was assessed using pulsed Doppler imaging of mitral inflow from an apical 4-chamber view. Parameters included isovolumic relaxation time (IVRT), early (E) and atrial (A) filling velocities, and myocardial performance index (MCPI). All image analyses were conducted in a blinded manner using the Prospect T1 software suite (Scintica).

**Skeletal Muscle Molecular and Functional Assays**

*Protein extraction and immunoblotting*

Protein was extracted from skeletal muscle tissue or cultured cells as previously described ^7^. Briefly, samples were homogenized in radioimmunoprecipitation assay (RIPA) buffer (Sigma-Aldrich, R0278) supplemented with Complete Mini Protease Inhibitor Cocktail (Sigma-Aldrich, 05892970001) and PhosSTOP Phosphatase Inhibitor Cocktail (Sigma-Aldrich, 4906845001). Tissue homogenization was performed using a TissueLyser (Qiagen, Hilden, Germany), followed by sonication with a Branson Sonifier (Thermo Fisher Scientific, SFX150). Lysates were centrifuged at 14,000 × g, and the supernatant was collected for downstream analysis.

Protein concentration was determined using the bicinchoninic acid (BCA) assay (Thermo Fisher Scientific, PI23225). Homogenates were diluted to a concentration of 2 µg/µL and mixed with 4× loading buffer. Equal amounts of protein (10–20 µg) were loaded onto 4–15% gradient SDS-polyacrylamide gels and resolved by electrophoresis. Proteins were transferred to nitrocellulose membranes and stained with Ponceau S (Sigma-Aldrich, P7170) to confirm consistent loading. Membranes were then blocked in 5% bovine serum albumin (BSA) and incubated overnight at 4°C with primary antibodies diluted in 5% BSA.

The following primary antibodies were used at 1:1,000: pAMPK^Thr172 (Cell Signaling, 2535S), total AMPK (2532), pACC^Ser212 (3661S), ACC (3676S), OXPHOS cocktail (Abcam, ab110413), PGC-1α (Millipore, AB3242), OPA1 (Abcam, ab42364), MFN2 (Cell Signaling, D1E9), pDRP1^Ser637 (Cell Signaling, 4867), pDRP1^Ser616 (3455), DRP1 (8570), FIS1 (Proteintech, 10956-1-AP), PINK1 (Novus, BC100-494), Utrophin (Leica, NCL-DRP2), γ-Sarcoglycan (Leica, G-SARC-CE), and β-Dystroglycan (DSHB, MANDAG2-7D11).

The following day, membranes were washed with TBST and incubated with horseradish peroxidase (HRP)-conjugated secondary antibodies (1:10,000; Cell Signaling, 7074S or 7076S). Chemiluminescent detection was performed using the ChemiDoc MP Imaging System (Bio-Rad, Mississauga, ON, Canada). Band intensities were quantified using Image Lab software and normalized to Ponceau S staining. For consistency across gels, a pooled internal control was included and used for normalization.

*Gene expression analysis by qPCR*

Total RNA was extracted from frozen skeletal muscle tissue using TRIzol reagent (Thermo Fisher Scientific, 15596018). Samples were homogenized in Lysing D Matrix tubes (MP Biomedicals, 6913-050) using the FastPrep-24 Tissue and Cell Homogenizer (MP Biomedicals). Following homogenization, samples were mixed with chloroform, shaken vigorously, and centrifuged at 12,000 × g according to the manufacturer’s instructions. The aqueous phase was recovered, and RNA was further purified using the Omega Bio-Tek total RNA purification kit (VWR, R6834-02). RNA concentration and purity were assessed using a NanoDrop 1000 Spectrophotometer (Thermo Fisher Scientific).

For cDNA synthesis, RNA was diluted to the appropriate concentration and reverse-transcribed using the High-Capacity cDNA Reverse Transcription Kit (Thermo Fisher Scientific, 4368814), following the manufacturer’s protocol.

Quantitative PCR (qPCR) was carried out using GoTaq qPCR Master Mix (Promega, A6002). Reactions were run in triplicate with 2 µg of input cDNA per reaction. The comparative CT (ΔΔCT) method ^8^ was used to calculate relative gene expression, and Rps11 was used as the housekeeping gene, as its expression was consistent across experimental conditions. qPCR primers (Sigma-Aldrich, St. Louis, MO, USA) used were as follows: *Rps11*: F – CGTGACGAACATGAAGATGC, R – GCACATTGAA TCGCACAGTC; *Ppargc1a*: : F – AGTGGTGTAGCGACCAAT, R – GGGCAATCCGTCTTCATCCA; *Map1lc3*: F – CACTGCTCTGTCTTGTGTAGGTTG, R – CACTGCTCTGTCTTGTGTAGGTTG; *Sqstm1*: F – CCCAGTGTCTTGGCATTCTT, R – A GGGAAAGCAGAGGAAGCTC; *Ulk1*: F – GCTCCGG TGACTTACAAAGCTG, R – GCTGACTCCAAGCCAAAG CA; *Gabra1*: F – CA TCGTGGAGAAGGCTCCTA, R – ATACAGCTGGCCCATGGTAG; *Pax7*: F – TTGGGGAACACTCCGCTGTGC, R – CAGGGCTTGGGAAGGGTTGGC; *Myf5*: F – TGAAGGATGGACATGACGGAC, R – TTGTGTGCTCCGAAGGCTGCT; *MyoD*: F – TCTGGAGCCCTCCTGGCACC, R – CGGGAAGGGGGAGAGTGGGG; *MyoG*: F – GGAATTCGAGGCATATTATGA, R – TCACATAAGGCTAACACCCAG; *Ctgf*: F – AGCTGGGAGAACTGTGTACG, R – GCCAAATGTGTCTTCCAGTC; *Col1a1*: F – ATGTTCAGCTTTGTGGACCT, R – CAGCTGACTTCAGGGATGT; *Cd68*: F - CCAATTCAGGGTGGAAGAAA, R - GAGAGAGACAGGTGGGGATG*, Lgal3*: F – CAACCATCGGATGAAGAACC, R – TTCCCACTCCTAAGGCACAC.

*Immunofluorescence Analysis*

Fiber typing and morphometric analysis of skeletal muscle were performed as previously described. Gastrocnemius (GAST) muscles were embedded in OCT and cryosectioned at −20°C into 10 µm-thick sections using a Leica cryostat ^9^. Sections were mounted on Superfrost Plus Gold slides (Thermo Fisher Scientific, 22-035813) and stored at −80°C until staining.

For immunofluorescence, sections were blocked in 10% goat serum diluted in 1% bovine serum albumin (BSA) for one hour at room temperature. Primary antibody incubation was then performed for two hours at room temperature using a cocktail diluted in 10% goat serum. The primary antibodies included myosin heavy chain type I (MyHCI; BA-F8, DSHB), myosin heavy chain type IIa (MyHCIIa; SC-71, DSHB), myosin heavy chain type IIb (MyHCIIb; BF-F3, DSHB), and laminin (L0663, Sigma-Aldrich). After several washes with phosphate-buffered saline (PBS), sections were incubated for one hour with a secondary antibody cocktail composed of Alexa Fluor-conjugated isotype-specific antibodies (IgG2b, IgG1, and IgM; Invitrogen) and an Alexa Fluor 647-conjugated anti-rat antibody (Jackson ImmunoResearch, 112-605-167). Following the final washes, sections were air-dried and mounted using ProLong Gold antifade reagent (Thermo Fisher Scientific, P36930).

Muscle sections were imaged using a Nikon 20× Plan Fluor 0.5 NA objective and a widefield photometric camera (Accu-Scope, Commack, NY, USA). Full muscle cross-sections were scanned, and regions enriched in slow oxidative and fast glycolytic fibers were identified based on myosin isoform staining. Morphological analysis of individual fibers, including cross-sectional area (fCSA) and minimum Feret diameter, was conducted by manually thresholding binary images. On average, 30 to 40 percent of each cross-section was analyzed, corresponding to over 200 fibers per sample.

To assess sarcolemmal membrane integrity, additional GAST sections were stained for endogenous mouse IgG. Sections were incubated overnight at 4°C with Alexa Fluor 488-conjugated goat anti-mouse IgG (A11029; Thermo Fisher Scientific), washed three times in PBS, and mounted with ProLong Gold. Images were acquired at 20× magnification using Nikon Elements software and analyzed using NIS Elements. IgG-positive regions were manually thresholded, and the extent of membrane permeability was expressed as a percentage of the total muscle cross-sectional area.

The localization and expression of utrophin in skeletal muscle sections were performed as previously described ^7^. Briefly, 10 µm cryosections were first incubated in a mouse-on-mouse (MOM) blocking reagent (BMK-2202, Vector Laboratories, Brockville, ON, Canada) diluted in 10% goat serum for 60 minutes at room temperature. Following the blocking step, the sections were incubated for 120 minutes with a primary antibody against utrophin (1:100; NCL-DRP2, Leica Biosystems, Concord, ON, Canada). Detection was achieved using a MOM biotinylated anti-mouse reagent followed by streptavidin amplification. To visualize muscle architecture, the slides were additionally stained with laminin (1:500, L0663, Sigma-Aldrich) for 120 minutes at room temperature, and then incubated with a 647-conjugated rat secondary antibody (1:500, 112-605-167, Jackson ImmunoResearch, West Grove, PA, USA). To mark synaptic regions, a fluorescently conjugated α-bungarotoxin (αBTX, 1:500, 13422, Thermo Fisher Scientific, Waltham, MA, USA) was used. Slides were thoroughly washed in PBS between each staining step and mounted with Prolong Gold antifade reagent (P366930, Thermo Fisher Scientific). Fluorescence intensity profiles were generated to quantify the synaptic versus extrasynaptic distribution of utrophin. Using NIS Elements AR 3.2 software (Nikon Instruments, Mississauga, ON, Canada), linear region-of-interest (ROI) traces were drawn across sarcolemmal αBTX-positive fibers to capture both synaptic and surrounding regions. A line width of five pixels was used to smooth out signal noise. Fluorescence intensity was plotted as a function of distance across the ROI.

For ɣ-sarcoglycan (ɣSG) and β-dystroglycan (βDG) localization, adjacent 10 µm sections were incubated in 10% goat serum blocking solution for 60 minutes at room temperature. Sections were then stained overnight at 4°C with primary antibodies against ɣSG (1:100, G-SARC-CE, Leica Biosystems) or βDG (1:100, MANDAG2-7D11, Developmental Studies Hybridoma Bank, Iowa City, IA, USA) in 1% BSA. On the following day, goat anti-mouse IgG Alexa Fluor 488 secondary antibody (1:500; A-11029, Thermo Fisher Scientific) was applied for one hour at room temperature. After additional PBS washes, laminin was reapplied (1:500, L0663, Sigma-Aldrich) and detected with a 647-conjugated anti-rat secondary antibody (1:500, 112-605-167, Jackson ImmunoResearch). Slides were mounted using Prolong Gold (P36930, Thermo Fisher Scientific) and imaged under identical settings for consistency in localization assessment.

*Histological preparations of skeletal and cardiac muscle sections*

Masson trichrome, Sirius Red, and hematoxylin and eosin (H&E) staining were performed according to manufacturer instructions. Stained sections were imaged by light microscopy at 10× magnification using Nikon Elements Microscopic Imaging Software (Nikon Instruments, Mississauga, ON, Canada). Masson trichrome images were analysed in NIS Elements, and Sirius Red–stained TIFF images were analysed in ImageJ (NIH). For Sirius Red quantification, colour channels were split and the green channel was thresholded to detect collagen. ROIs were drawn to outline the total muscle and to isolate artefact-free regions. ROIs and percent collagen values were then combined to generate a weighted percent collagen area, normalized to the fraction of muscle represented by the analysed ROIs.

*Whole-mount muscle preparation, immunofluorescence, and confocal microscopy*

The immunohistochemical labeling of the pre- and postsynaptic components was adapted from previous methods ^10^. Whole ETA muscles were muscles were carefully dissected in oxygenated Ringer’s Solution [110 mM NaCl, 5 mM KCl, 1 mM MgCl_2_, 25 mM NaHCO_3_, 2 mM CaCl_2_, 11 mM glucose, 0.3mM glutamic acid, 0.4 mM glutamine, 5 mM BES (N,N-Bis(2-hydroxyethyl)-2-aminoethanesulfonic acid sodium salt, 0.036 mM choline chloride, and 4.34 x 10^−7^ mM cocarboxylase] and pinned in a Sylgard-coated 10 mm Petri dish. To fix the muscles, samples were treated with 4% PFA at room temperature for 10 mins and subsequently permeabilized with cold methanol at -20 °C for 6 mins. To block nonspecific labeling, the muscles were incubated with 10% normal goat serum in PBS containing 0.1% Triton X-100 for 60 mins at room temperature. Motoneuron axons were labeled using anti-neurofilament M (anti-neurofilament M; 1:50; Developmental Studies Hybridoma Bank, Iowa City, IA, USA; 2H3), and nerve terminals were labeled with anti-synaptic vesicular protein 2 (mouse IgG1 anti-synaptic vesicular protein 2, 1:100; Developmental Studies Hybridoma Bank, Iowa City, IA, USA; SV2) overnight at 4 °C. On the following day, the muscles were incubated with secondary antibodies, specifically goat anti-mouse IgG Alexa-594 (1:500, Jackson ImmunoResearch, West Grove, PA, USA) for 60 mins. Postsynaptic acetylcholine receptors were labeled with Alexa-488-conjugated ⍺-bungarotoxin (1:500, Thermo Fisher Scientific, Waltham, MA, USA; B13423) for 60 mins. All antibody incubations were carried out in PBS containing 0.1% Triton X-100 and 2% normal goat serum at room temperature. After each step, the muscles were rinsed three times in PBS containing 0.01% Triton X-100 for 5 mins each. Finally, the samples were mounted in Prolong Gold antifade reagent (Thermo Fisher Scientific, Waltham, MA, USA; P36930), and images were captured using confocal microscopy (x60, 1.4 NA oil immersion; Nikon Instruments, Mississauga, ON, Canada).

*Preparation of permeabilized fiber bundles*

Following cervical dislocation of the mouse, the QUAD muscle was rapidly dissected and placed in an ice-cold Biopsy Preservation Solution (BIOPS) buffer (50 mM K-MES, 7.23 mM K_2_EGTA, 2.77 mM CaK_2_EGTA, 20 mM imidazole, 20 mM taurine, 5.7 mM ATP, 14.3 mM phosphocreatine, and 6.56 mM MgCl_2_, pH of 7.1). The immersed muscle was further dissected to clear connective tissue and fat and then portioned into smaller muscle bundles. Fiber bundles were separated and placed in a BIOPS containing saponin (Sigma-Aldrich, St. Louis, MO, USA; S7900) and 2,4-dinitrochlorobenzene (CDNB; Sigma-Aldrich, St. Louis, MO, USA; D1529; 35 μM) at 4^o^C for 30 mins. After the permeabilization step, the muscle fibers were washed in Buffer Z (105 mM K-MES, 30 mM KCl, 10 mM KH_2_PO_4_, 5 mM MgCl_2_, 1 mM EGTA, 5 mg mL^-1^ BSA, pH 7.4) at 4 °C for 15 mins.

*Mitochondrial respiration and H_2_O_2_ emission measurement in permeabilized muscle fibers*

High-resolution respirometry was conducted using the Oxygraph-2k system (Oroboros Instruments, Innsbruck, Austria) at a temperature of 37 °C, while maintaining [O_2_] at approximately 250 - 400 μM. Prior to introducing 2 mL of Buffer Z, horseradish peroxidase (4 U mL^-1^), superoxide dismutase (30 U mL^-1^; Sigma-Aldrich, St. Louis, MO, USA), and Amplex Red (10 μM; Sigma-Aldrich, St. Louis, MO, USA; A36006), the chambers were volume and air calibrated. To calibrate the fluorometric sensor, five titrations of 0.1 μM H_2_O_2_ were serially added. Once the permeabilized muscle sample (2 - 3 mg) was added, the system was allowed to equilibrate until the O_2_ slope traces reached a steady-state. The experiment proceeded with the addition of pyruvate (5 mM; Sigma-Aldrich, St. Louis, MO, USA; S8636) and malate (2 mM; Sigma-Aldrich, St. Louis, MO, USA; M8304), followed by ADP (5 mM; Sigma-Aldrich, St. Louis, MO, USA; A2754), glutamate (5 mM; Sigma-Aldrich, St. Louis, MO, USA; G1626), Cytochrome C (10 mM; Sigma-Aldrich, St. Louis, MO, USA; C2506), and succinate (10 mM; Sigma-Aldrich, St. Louis, MO, USA; S2378). During the experiment, Amplex-Red-derived fluorescence was measured simultaneously with O_2_ consumption using the O2k-Fluo LED2-Module (Oroboros Instruments, Innsbruck, Austria). To account for variations in muscle size, values were normalized based on the wet muscle weights. Data collection and analysis were performed using DatLab (V.7.4, Oroboros Instruments, Innsbruck, Austria).

*Transmission electron microscopy evaluation of intermyofibrillar mitochondria*

Freshly dissected TA muscles were promptly fixed in a 2% (v/v) glutaraldehyde solution with 0.1 mol L⁻¹ sodium cacodylate buffer (pH 7.4). The fixed samples were then embedded and sectioned into thin slices (70 nm) using an ultramicrotome (Ultracyte E; Reichert, Vienna, Austria). These sections were placed on Cu/Pd grids and stained with uranyl acetate for 5 minutes, followed by lead acetate for 2 mins. Mitochondria were identified based on the presence of a complete or partial outer membrane and visible cristae. Mitochondria that exhibited more than 20% swelling or displayed abnormal cristae characteristics, such as paracrystalline inclusions, linearized and angular features, concentric cristae, compartmentalization, outer membrane lesions, and giant mitochondria, were documented, similar to previously described in the literature ^11,12^.

In vitro metabolic measurements of primary myotubes.

Oxygen consumption rates (OCR) and extracellular acidification rates (ECAR) were determined in XFe24 plates using the Seahorse XFe24 Analyzer (Agilent, Mississauga, ON, Canada). DMD patient-derived myoblasts were seeded at 40,000 - 60,000 cells per well in triplicates with 250 µL of culture medium, for 24 hrs prior to performing the assay. OCR was evaluated using oligomycin A (1 µM; Sigma-Aldrich, St. Louis, MO, USA; 75351), carbonyl cyanide-p-trifluoromethoxyphenylhydrazone (2.0 µM; FCCP; Sigma-Aldrich, St. Louis, MO, USA; C2920), rotenone (0.5 µM; Sigma-Aldrich, St. Louis, MO, USA; R8875) and antimycin A (0.5 µM; Sigma-Aldrich, St. Louis, MO, USA; A8674). All values were normalized to protein concentration as determined by homogenization in 50 µL per well of RIPA lysis buffer (Thermo Fisher Scientific, Waltham, MA, USA; 89901) and subsequent BCA Protein Assay Kit analysis (Thermo Scientific, Waltham, MA, USA; 23225). Experiments were performed in technical triplicates where each independent triplicate was included for statistical analysis. OCR-related outcomes were calculated as per manufacture instruction.

**REFERENCES**

1. Flanigan KM, Ceco E, Lamar KM, Kaminoh Y, Dunn DM, Mendell JR *et al.* LTBP4 genotype predicts age of ambulatory loss in duchenne muscular dystrophy. *Ann Neurol* 2013;**73**:481–488.

2. Mázala DAG, Novak JS, Hogarth MW, Nearing M, Adusumalli P, Tully CB *et al.* TGF-β-driven muscle degeneration and failed regeneration underlie disease onset in a DMD mouse model. *JCI Insight* 2020;**5**.

3. Myers RW, Guan H-P, Ehrhart J, Petrov A, Prahalada S, Tozzo E *et al.* Systemic pan-AMPK activator MK-8722 improves glucose homeostasis but induces cardiac hypertrophy. *Science* 2017;**357**:507–511.

4. Ng SY, Mikhail AI, Mattina SR, Mohammed SA, Khan SK, Desjardins EM *et al.* AMPK regulates the maintenance and remodelling of the neuromuscular junction. *Mol Metab* 2025;**91**:102066.

5. Mattina SR, Ng SY, Mikhail AI, Stouth DW, Jornacion CE, Rebalka IA *et al.* Volitional exercise elicits physiological and molecular improvements in the severe D2.mdx mouse model of Duchenne muscular dystrophy. *J Physiol* 2025;**0**:1–19.

6. Hamstra SI, Geromella MS, Tiidus P, Klentrou P, MacPherson REK, Fajardo VA. Subtherapeutic lithium supplementation causes physiological eccentric cardiac hypertrophy in young-adult wild-type male mice. *Physiol Rep* 2025;**13**:e70299.

7. Ng SY, Mikhail AI, Mattina SR, Manta A, Diffey IJ, Ljubicic V. Acute, next-generation AMPK activation initiates a disease-resistant gene expression program in dystrophic skeletal muscle. *FASEB J* 2023;**37**:e22863.

8. Schmittgen TD, Livak KJ. Analyzing real-time PCR data by the comparative CT method. *Nat Protoc* 2008;**3**:1101–1108.

9. Bloemberg D, Quadrilatero J. Rapid determination of myosin heavy chain expression in rat, mouse, and human skeletal muscle using multicolor immunofluorescence analysis. *PLoS One* 2012;**7**.

10. Villarroel-Campos D, Schiavo G, Sleigh JN. Dissection, in vivo imaging and analysis of the mouse epitrochleoanconeus muscle. *J Anat* 2022;**241**:1108–1119.

11. Vincent AE, Ng YS, White K, Davey T, Mannella C, Falkous G *et al.* The Spectrum of Mitochondrial Ultrastructural Defects in Mitochondrial Myopathy. *Sci Rep* 2016;**6**:1–12.

12. vanLieshout TL, Stouth DW, Hartel NG, Vasam G, Ng SY, Webb EK *et al.* The CARM1 transcriptome and arginine methylproteome mediate skeletal muscle integrative biology. *Mol Metab* 2022;**64**:101555.
