# Supplementary material for: Direct AMPK Activation Confers Mutation‐Independent Therapeutic Benefit in Duchenne Muscular Dystrophy
Source: J Cachexia Sarcopenia Muscle. 2026 Feb 4;17(1):e70200. doi: 10.1002/jcsm.70200 (PMC12872333; doi:10.1002/jcsm.70200)
Supplement: Supplementary file 2 — Table S1: Patient‐derived myotube characteristics. Figure S1: Chronic MK treatment does not exacerbate the dystrophic cardiac phenotype in D2.mdx animals. (A) Representative M‐mode in vivo echocardiography images in WT Veh, D2.mdx Veh, and D2.mdx MK mice during Week 7 of Veh or MK treatment. (B) Summary of predicted cardiac morphology metrics (millimetres; mm), including left ventricle anterior wall thickness (LVAW), left ventricle internal diameter (LVID) and left ventricle posterior wall thickness (LVPW) during systole and diastole. (C) Heart mass relative to body mass. (D) Relative ventricular septum wall thickness during systole and diastole in WT Veh, D2.mdx Veh, and D2.mdx MK mice. (E) Fractional shortening (FS) and ejection fraction (EF) expressed as percentages. (F) Cardiac output (mL min−1) in WT Veh‐ and Veh‐ and MK‐treated D2.mdx animals. (G) Representative mitral valve E and A wave images in WT Veh, D2.mdx Veh and D2.mdx MK animals. (H–N) Summaries of mitral valve metrics, including peak velocity (H) and deceleration (I) of E waves, peak velocity (J) and acceleration (K) of A waves, E/A peak velocity ratio (L), as well as isovolumetric (IV) contraction and relaxation times (M), and myocardial performance index (N). Graphical summaries show individual data points and group means with SEM (n = 5–6). (O–P) Sirus Red and haematoxylin and eosin staining on cardiac sections from WT Veh, D2.mdx Veh and D2.mdx MK mice. Scale bar: 100 μm. (Q) Quantification of cardiac fibrosis area (%) in WT Veh, D2.mdx Veh and D2.mdx MK animals. Statistical significance indicated by #, p < 0.05 versus Veh‐treated D2.mdx animals. Figure S2: Repeated MK treatment enlarges postsynaptic features of the neuromuscular junction (NMJ) in skeletal muscles of D2.mdx animals. (A) Confocal IF microscopy images of NMJs in the epitrochleoanconeus muscle of Veh‐treated WT and Veh‐ or MK‐treated D2.mdx animals. Presynaptic morphology is visualized using labels for neurofilament M and synaptic v [file JCSM-17-e70200-s001.docx]

**SUPPLEMENTAL MATERIAL**

| **Cell line** | **Sex** | ***DMD* mutation** | **Age at biopsy (years)** |
| --- | --- | --- | --- |
| *DMDΔ44* | Male | Exon 44 deletion | 1 |
| *DMDΔ45* | Male | Exon 45 deletion | 3 |
| *DMDΔ22-29* | Male | Exon 22-29 deletion | 2 |
| *DMDΔ45-52* | Male | Exon 45-52 deletion | 2 |
| CTRL | Male | N/A | 3 |

**Table 1: Patient-derived myotube characteristics.**

DMD, Duchenne muscular dystrophy. CTRL, Control.

**Figure S1:** **Chronic MK treatment does not exacerbate the dystrophic cardiac phenotype in D2.mdx animals. (A)** Representative M-mode in vivo echocardiography images in WT Veh, D2.mdx Veh, and D2.mdx MK mice during week 7 of Veh or MK treatment. (**B**) Summary of predicted cardiac morphology metrics (millimetres; mm), including left ventricle anterior wall thickness (LVAW), left ventricle internal diameter (LVID), and left ventricle posterior wall thickness (LVPW) during systole and diastole. **(C)** Heart mass relative to body mass. **(D)** Relative ventricular septum wall thickness during systole and diastole in WT Veh, D2.mdx Veh, and D2.mdx MK mice. **(E)** Fractional shortening (FS) and ejection fraction (EF) expressed as percentages. **(F)** Cardiac output (ml min^-1^) in WT Veh- and Veh- and MK-treated D2.mdx animals. **(G)** Representative mitral valve E and A wave images in WT Veh, D2.mdx Veh, and D2.mdx MK animals. **(H-N)** Summaries of mitral valve metrics, including peak velocity **(H**) and deceleration **(I)** of E waves, peak velocity **(J)** and acceleration **(K)** of A waves, E/A peak velocity ratio **(L)**, as well as isovolumetric (IV) contraction and relaxation times **(M)**, and myocardial performance index **(N)**. Graphical summaries show individual data points and group means with SEM (n = 5–6). **(O-P)** Sirus Red and hematoxylin and eosin staining on cardiac sections from WT Veh, D2.mdx Veh, and D2.mdx MK mice. Scale bar: 100 μm. **(Q)** Quantification of cardiac fibrosis area (%) in WT Veh, D2.mdx Veh, and D2.mdx MK animals. Statistical significance indicated by #, p < 0.05 versus Veh-treated D2.mdx animals.

**
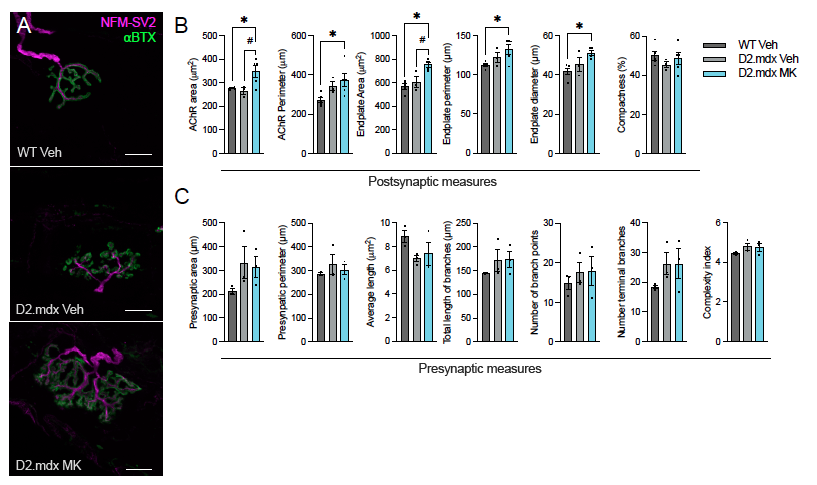
**

**Figure S2: Repeated MK treatment enlarges postsynaptic features of the neuromuscular junction (NMJ) in skeletal muscles of D2.mdx animals. (A)** Confocal IF microscopy images of NMJs in the epitrochleoanconeus muscle of Veh-treated WT, and Veh- or MK-treated D2.mdx animals. Presynaptic morphology is visualized using labels for neurofilament M and synaptic vesicle 2 (NFM+SV2, magenta), and AChRs at the motor endplate are presented using ⍺BTX (green). Scale bar: 20 μm. **(B-C)** Pre- and postsynaptic NMJ morphology metrics for all experimental groups, derived from NMJmorph analysis. Graphical summaries show individual data points and group means with SEM (n = 4–5). Statistical significance indicated by #, p < 0.05 versus Veh-treated D2.mdx animals.

**
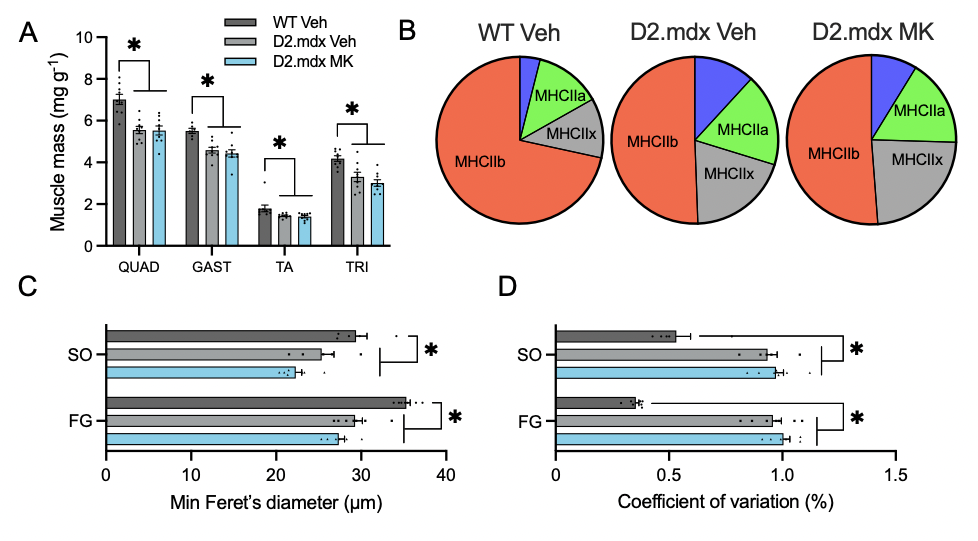
**

**Figure S3. Skeletal muscle mass and myofiber morphology analyses in D2.mdx mice following MK treatment. (A)** Muscle mass of quadriceps (QUAD), gastrocnemius (GAST), TA, and triceps (TRI) from Veh- and MK-treated animals. Values are expressed relative to body mass (mg g^-1^). **(B)** Circle graphs of myosin heavy chain fiber type distributions in GAST muscles from WT Veh, D2.mdx Veh, and D2.mdx MK mice. **(C-D)** Graphical summaries of average minimum Feret’s diameter (µm) **(C)** and fiber size variation (expressed as coefficient of variation percentage) **(D)** in the slow oxidative and fast glycolytic regions of GAST muscles. Data are presented as fold changes relative to the WT Veh group. Graphs display individual data points, group means (bars), and SEM. n = 8–10. Statistical significance is denoted as: *, p < 0.05 vs. WT Veh; #, p < 0.05 vs. D2.mdx Veh.


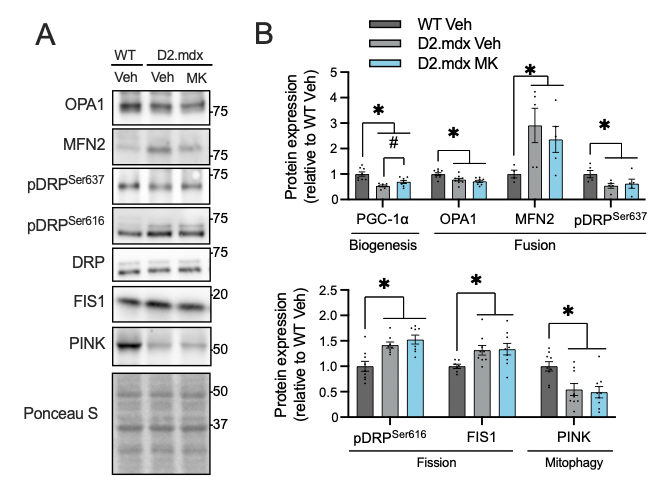


**Figure S4. Mitochondrial dynamic proteins are disrupted in D2.mdx muscle but unaffected by MK treatment. (A)** Representative Western blots of optic atrophy 1 (OPA1), mitofusin-2 (MFN2), serine (Ser) 637-phosphorylated DRP (pDRP^Ser637^), Ser616-phosphorylated dynamin-related protein (pDRP^Ser616^), DRP, fission protein 1 (FIS1), and PTEN-induced kinase 1 (PINK) in TA muscle lysates from Veh-treated WT, and Veh- or MK-treated D2.mdx mice. Ponceau S staining is shown as a loading control. Protein ladder markers (kDa) are indicated. **(B)** Graphical summaries of protein levels related to mitochondrial fusion, fission, and mitophagy in Veh-treated WT, and Veh- or MK-treated D2.mdx mice. Data are presented as fold changes relative to the WT Veh group. Graphs display individual data points, group means (bars), and SEM. n = 8–10. Statistical significance is denoted as: *, p < 0.05 vs. WT Veh; #, p < 0.05 vs. D2.mdx Veh.

**
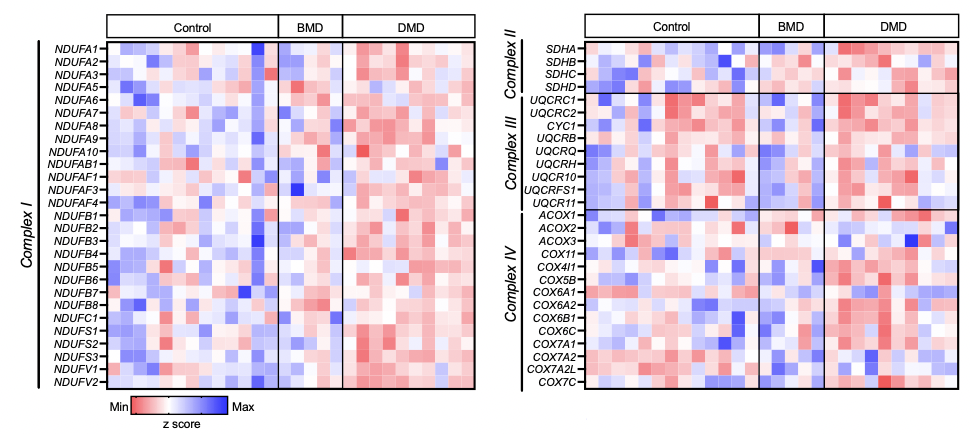
**

**Figure S5. DMD patient skeletal muscle biopsies show downregulation of mitochondrial complex I genes.** Heatmap showing expression of mitochondrial ETC genes curated from the Gene Ontology (GO) category "Mitochondrial electron transport, NADH to ubquinone; succinate to ubiquinone; cytochrome c to oxygen, ubiquinol to cytochrome c" (GO:0006120; GO: 0006121; GO: 0006123; GO: 0006122) in skeletal muscle biopsies from healthy controls, Becker muscular dystrophy (BMD), and DMD patients from the GSE3307 dataset.
